# 3D reactive inkjet printing of aliphatic polyureas using in-air coalescence technique†

**DOI:** 10.1039/d1ra07883f

**Published:** 2022-01-25

**Authors:** Maciej Zawadzki, Krzysztof Zawada, Sebastian Kowalczyk, Andrzej Plichta, Jan Jaczewski, Tomasz Zabielski

**Affiliations:** Faculty of Chemistry, Warsaw University of Technology Noakowskiego 3 00-664 Warsaw Poland mzawadzki@ch.pw.edu.pl +48 (22) 234 7475; Zdalny Serwis sp z o.o. Wysowska 12 02-928 Warsaw Poland; AVICON Advanced Vision Control Jerozolimskie 202 Warsaw Poland

## Abstract

An in-flight coalescence reactive inkjet printer has been developed to facilitate the in-air collision of two reactive microdroplets. This way precise volumes of reactive inks can be mixed and subsequently deposited on the substrate to produce the desired product by polymer synthesis and patterning in a single step. In this work, we validate the printer capabilities by fabrication of a series of 3D structures using an aliphatic polyurea system (isophorone diisocyanate IPDI and poly(propylene glycol) bis(2-aminopropyl ether) PEA-400). The influence of temperature and ink ratio on the material properties has been investigated. An increase in both IPDI and temperature facilitates the production of materials with higher Young's Modulus *E* and higher ultimate strength *U*. The possibility of printing different materials *i.e.* ductile (*U* = 2 MPa, *ε*_B_ = 450%), quasi-brittle (*U* = 14 MPa, *ε*_B_ = 350%), and brittle (*U* = 10 MPa, *ε*_B_ = 11%) by varying the printing process parameters using one set of inks has been presented. The anisotropy of the material properties arising from different printing directions is at the 20% level.

## Abstract 2

In flight coalescence of polyurea inks in different volume ratios produces materials with varied degrees of elasticity.

## Graphical abstract

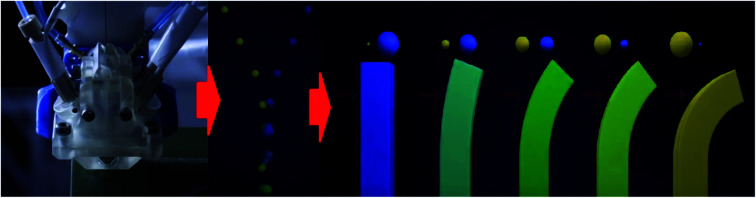

## 1. Introduction

3D printing or additive manufacturing is a promising technology with the potential to change almost every human enterprise.^1^ The world of 3D printing can be roughly split into three segments. The first segment consists of fusing previously prepared material and is represented by Fused Deposition Modelling (FDM),^2^ Selective Laser Sintering or Melting (SLS, SLM)^3^ and HP MultiJet Printing.^4^ The second segment gathers technologies where previously prepared resins are cured by light, usually UV: Stereolithography (SLA),^5^ Continuous Liquid Interface Production (CLIP),^6^ PolyJet Printing^7^ and Volumetric Additive Manufacturing (VAM).^8^ The third segment characterizes technologies were material cures due to mixing of two or more reactive inks: Reactive Additive Manufacturing (RAM)^9^ and Reactive Inkjet Printing (RIJ).^10^

Inkjet printing is a commonly used technology in which precise volumes of inks are deposited on a substrate. Reviews discuss its appeal as a low-cost fabrication technique and its successful usage for several applications beyond its wide use in graphic printing.^11^ Most chemical reactions are carried out in liquid solvents thus the ability to deposit precise picolitre volumes of solutions in the desired place is exciting to any chemist. In this way, many different chemical reactions can be studied as well as utilized. Inkjet printing realizes a mask-free, cost-effective, and rapid method to pattern functional materials with high spatial resolution. However, reactive inkjet printing applications for additive manufacturing are scarce due to the requirement of low fluid viscosity which limits the variety and performance of the printed materials.^12^

The Reactive inkjet printing, similarly, to Reaction Injection Molding (RIM),^13^ aims at utilizing a chemical nature of different chemical formulations to react and produce desired materials. It eliminates the viscosity issue as well as other problems in inks stability before deposition.^14,15^ By storing reactive inks in separate reservoirs and subsequently depositing them on the substrate to synthesize a variety of materials with a wide spectrum of possible material properties.^16,17^ However, this requires a multistep process in short time scales with precise control of the environment, and products are often distorted due to Marangoni flow.^18^ Additionally, the reaction product can form an impenetrable barrier to substrates resulting in unreacted ink trapped within the print. For these reasons only relatively slow reacting inks and/or porous substrates are selected to limit the complexity of the printing process.^19,20^ In this way materials with properties widely different than in standard 3D printing can be achieved.

Reactive inks can be contacted within the printhead such as in the RAM process, but this approach would result in quick dispenser clogging due to an increase of ink viscosity because of chemical reactions taking place in the mixture.^21^ Droplets can also be coalesced in-flight to form a transient micro-reaction vessel for chemical synthesis.^22^ In such micro-reactors substrate mixing is facilitated by the kinetic energy of the droplet collision and impact onto the surface. Thus, reactive inkjet printing with in-flight droplet coalescence offers new possibility of realizing the printing process.^23^ This method has been reported in the literature by Stringer *et al.* for the formation of hydrogels,^24^ conductive polyaniline inks,^25^ poly(3,4-ethylene dioxythiophene) polystyrene sulfonate (PEDOT:PSS) system^26^ or covalent organic frameworks.^27^

In this study, we aim to showcase the possibility of micro-reactive inkjet printing to produce 3D structures. We have developed a micro-reactive inkjet printer capable of sustaining droplet collisions by online droplet parameters control and jetting parameters correction. A simple polyurea system has been chosen on the basis of rapid polymerization even in ambient conditions. This model system allowed for examination of process parameters and ink ratios impact on the material properties of obtained materials.

## 2. Experimental section

### 2.1. Reactive inkjet printing system

**Fig. 1:**
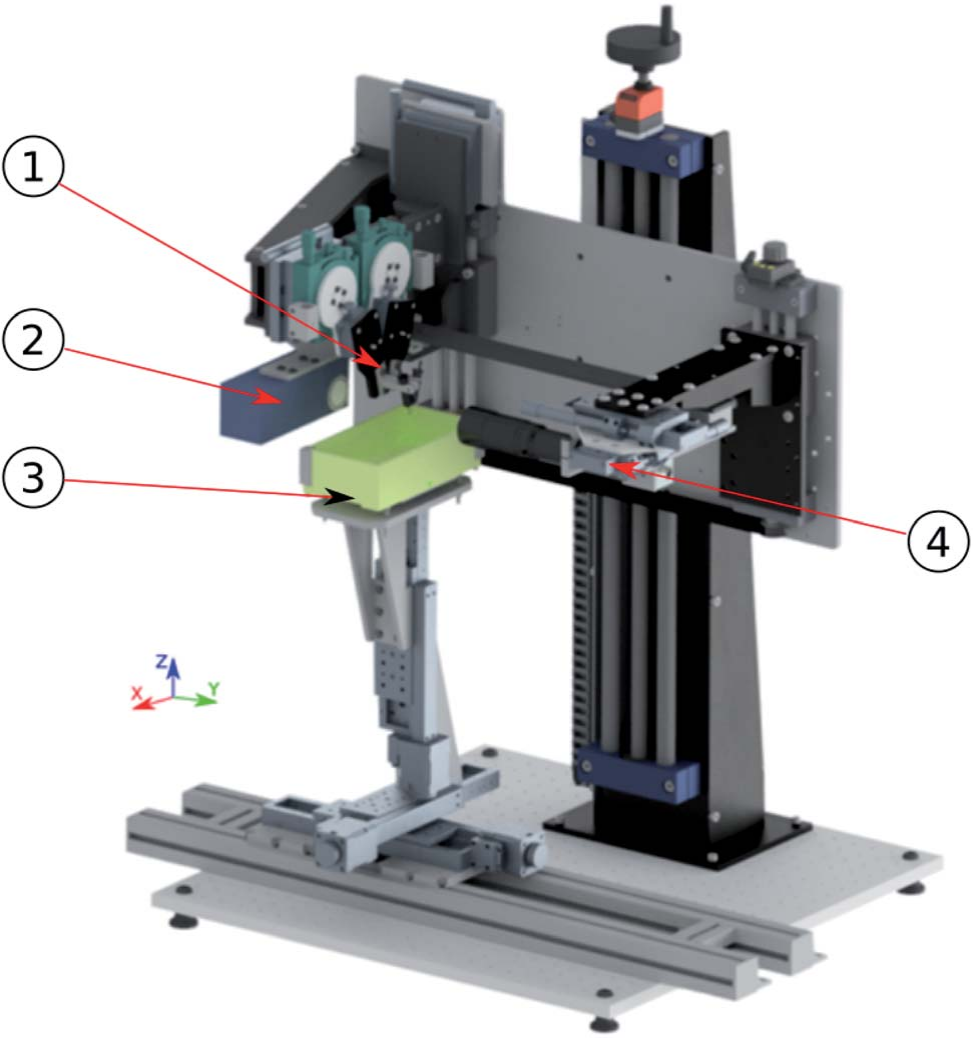
Designed and manufactured micro-reactive printer. 1-printing nozzles, 2-strobe, 3-printing table, 4-camera.

The schematic diagram of the developed micro-reactive inkjet printer is presented in Fig. S1.† In Fig. 1 a mechanical mounting was designed and manufactured to align all necessary components is presented.

### 2.2. Printer setup

The laboratory stand is based on a 15 mm aluminum optical breadboard with M6 tapped holes in a 25 mm grid. Both, the printing stage and optical-printing unit, are bolted to the base. Optical-printing unit is mounted on Igus drylin® SHT linear module J4Pz that allows the coarse regulation of system height. This allows adjusting the distance from the optical-printing assembly to the printing table used. The optical-printing unit consists of an optical system and printing system. Both are mounted on an aluminum plate bolted to the J4Pz joint. Optical system assembly is mounted on a series of linear guides. The J5Pz joint realized on two Igus drylin® SHT linear modules, coupled together with a toothed belt, allows the fine height adjustment between the optical axis and printing nozzles. Joint J7Px is realized on Standa 7T175-50 micrometric stage and provides a precise x regulation of nozzles in the optical axis, and as a result, droplets collision point in the camera image. Joint J6Py, also realized on Standa 7T175-50 micrometric stage, is used to adjust a droplet collision plane to the focusing plane of the lens, and thus acquire a sharp image. Joints J8Px, J1Py, J12Pz, realized on Standa 7T167S-25 micrometric stage, allow precise x, y, and z fine adjustment of the mutual linear position of the nozzles. The angle between nozzles (and thus collision angle), is adjusted using Standa precision rotary stages 7R129 J9Ry and J13Ry. Precise goniometers Standa 7G174-30 (J10Rx, J14Rx) are used to adjust the nozzles to be coplanar, which is necessary for droplet collisions to occur.

### 2.3. Inkjet dispensing setup

The ink dispensing setup has been purchased from Microdrop Technologies GmbH and consists of two dispenser heads (MD-K-140, 70 μm diameter, heated nozzle tip) and a control unit for two dispensers with pulse modulation and pressure control (MD-E-3021-131) connected to a specially designed process control unit.

### 2.4. Optical system

The optical system aims to acquire the images of flying droplets, to determine their velocity vector and position on the collision plane. These values are then used to determine the collision parameters and to control the printing process.

The optical system is based on a Basler acA2500-60 μm area scan camera, VS Technology VS-THV2-80/S telecentric lens, and LED stroboscope (specially designed for this application by AVICON company). The lens is characterized by a magnification of 2.0 and a long working distance of 80 mm. The optical resolution of the system is 2.4 μm/px allowing precise measurements of the droplets. To determine the velocity vector of each droplet, it is necessary to acquire two images, with a known delay and then locate the position of the droplets in the image. For research purposes, up to four images can be taken to analyze the collision process. Because of droplets' velocity (up to 8 m s^−1^) and small field of observation, the delay between images is in the range of tens to hundreds of microseconds. The ultrafast stroboscope is used to acquire multiple exposure image. It is designed to emit high-intensity flashes with a duration in the range of 150–400 ns, which is crucial for obtaining sharp, images without motion blur.

**Fig. 2:**
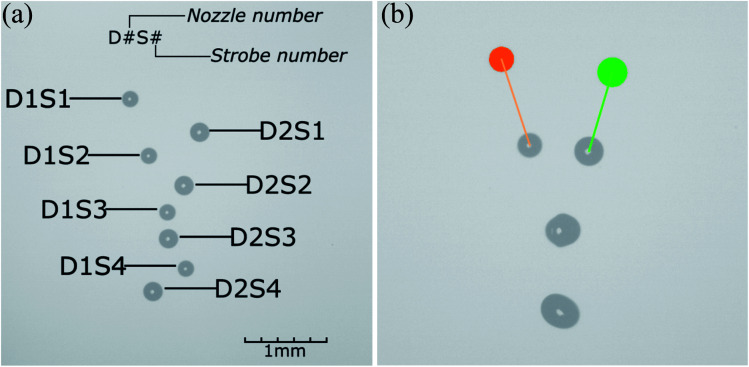
Images acquired with quadruple strobing during the droplet generation: (a) before trigger time correction; (b) after trigger time correction.

Fig. 2A shows an image acquired on the laboratory device. It is strobed four times, with equal delays between flashes. Smaller droplets are emitted by the first nozzle, while larger ones are emitted by the second one. The collision and mixing have not occurred - droplets passed each other. Acquired images are then analyzed with the process control system.

### 2.5. Process control system

The control system is based on a personal computer and a dedicated, custom-designed, microcontroller-based controller (Fig. S2†). PC, working under Microsoft Windows 10 OS, is responsible for image acquisition and analysis, calculation of process timing, and communication with the laboratory printer components. The main application is written in Python. Image analysis is necessary to calculate droplet velocity vector, position, and diameter and is realized using the Adaptive Vision Library software package.

Fig. 2B shows the droplets' positions and diameters (orange and green circles) and velocity vectors (orange and green line). The process parameters led to a collision (visible in the middle of the image) with full droplet coalescence.

Determined speed vectors, droplet positions, and sizes are used to calculate the Webber scalar and X parameter of collision. These two are used to calculate the desired delay between droplets generation. The controlled process parameter is a delay between the nozzles' activation. This parameter, together with the image acquisition timing, is sent to the μC controller using RS232. The μC controller is based on the STM32F722 ZE device. It was chosen because of multiple timers and over 200 MHz core operation frequency capability. The 200 MHz time base is used to generate strobe signals with nanosecond timing accuracy. The cascade timer connection and ultrafast circular DMA, used for modifying the timers registers, allowed precise and wide time-range control signals generation (Fig. S3† and Table 1). Piezoelectric printing nozzles from Microdrop company are used together with Microdrop controller. The PC application is used to adjust nozzles parameters such as heating temperature, pressure, piezo pulse voltage, and time, using commands sent to Microdrop controller *via* RS232. Microdrop controller's digital inputs are used for timing the nozzle activation. The signal is delivered from μC controller high-speed output. The ink parameters were measured by Anton Paar DMA4500M densimeter and Brookfield Ametek DVNext rheometer.

**Table 1:** Image acquisition parameter range and resolution

Parameter	Value range	Resolution
*t*_1_	−20 000 μs–20 000 μs	1 μs
*t*_2_	10 μs–5000 μs	1 μs
*T*_b_	150 ns–2000 ns	10 ns
*t*_3_, *t*_4_, *t*_5_	10 μs–5000 μs	1 μs
Number of strobe flashes	1–4	NA
Printing frequency	1–1000 Hz	1 Hz

### 2.6. Light source

The project required the development of an ultra-fast high-current strobe. Due to the short exposure time, the light source must have high instantaneous power. At the same time, the device works as a backlight, therefore an appropriate working area and high homogeneity of light are required. The block diagram of the strobe is presented in Fig. S4†

The strobe is triggered by a signal from the controller. To ensure the maximum level of synchronization with the controller and nozzle drivers, components with very low rise/fall times and low signal propagation time were used. Additionally, the power section is based on very fast MOSFET transistors. The dynamics of the MOSFET gate drivers and dead times have been selected in such a way as to minimalize the propagation time in the entire system. The delay time between the trigger signal and optical output is approx. 50–150 ns with an output power of 250–850 W. The strobe works with a switch-on time of 150–2000 ns. An important aspect is that the light signal should be as close as possible to the input signal. The closest possible electrical response of the system does not guarantee an analogous light response. Since a real LED is not a perfect object there are parasitic effects on it. As a result of, among others parasitic capacitance, the LED is still on for some time despite being turned off. The level of light in this phenomenon is lower than when the LED is on, and it fades over time. However, with such a precise and fast application, this problem is visible. The lighting time of the LED after switching off can be many times longer than the time of switching on. To eliminate this problem, a carrier sweep-out circuit has been used in the power section. With very steep slopes of the current waveform (high system dynamics), it was necessary to include additional protection in the power section.

The device can only work in strobe mode. To protect the device against permanent switching on, an internal microcontroller was used to control the strobe switching time. Additionally, it is possible for the system to react to the input edge and light output can be controlled by an internal controller. This time can be programmed *via* the RS-232 interface.

To ensure an appropriate working area, an LED made-in COB (Chip-on-Board) technology was used. The illuminating surface has the shape of a circle with a diameter of 9 mm. Homogeneity of the background was ensured using appropriate collimating optics.

### 2.7. Printing process

To realize the printing process a nozzles activation must be synchronized with a table movement. The G-code, created using an open slicer software Slic3R, is then sent to the xyz positioning stage controller. After interpretation of commands, the stepper motors are driven. During the table movement, the trigger signal for droplet generation is sent to the μC controller, with set pulse/mm resolution.

### 2.8. Model inks

**Fig. 3:**
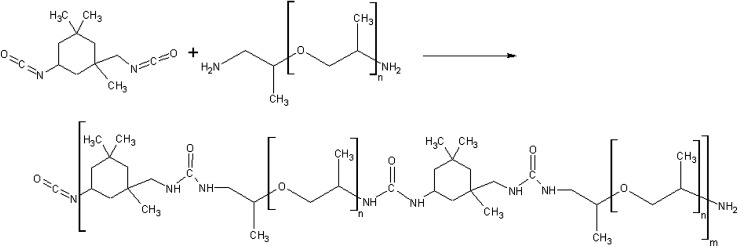
Reaction scheme for the formation of polyurea from the reactants.

**Fig. 4:**
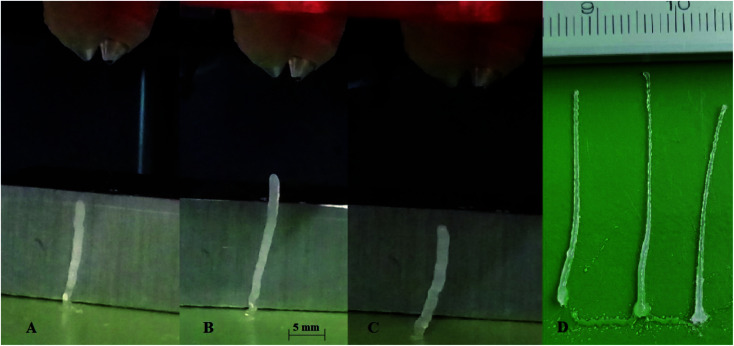
Pillars printed using IPDI + PEA 400 system with different IPDI excesses: (A) *R*^E^ = 5.6%; (B) *R*^E^ = −4%; (C) *R*^E^ = −12%. The collision point is located 4 mm from the dispensers and 32 mm from the surface. (D) full length pillars printed from IPDI + Jeffamine T-403 system.

From the available literature^28^ a diisocyanate isophorone IPDI (Sigma-Aldrich 98%, mixture of isomers) and poly(propylene glycol) bis(2-aminopropyl ether) with and number average molar mass of *M̄*_n_ = 430 g mol^−1^ PEA 400 (Sigma-Aldrich) system was selected. As a result of mixing and subsequent reaction polyurea polymer is produced (Fig. 3). The selection was based on short cure time allowing for pillar formation without catalyst addition as in the case of polyurethanes (Fig. 4). The IPDI was distilled under vacuum and stored under argon. The PEA 400 was dried using molecular sieves 3A. Methanol (Stanlab, 99%) was used to clean the dispensers due to its ability to dissolve both IPDI + PEA 400 as well as IPDI + water polymerization products.

### 2.9. Dispensing parameters

Jetting of droplets without satellite droplets is possible with Ohnesorge number greater less than 1 and greater than 0.1, or Ohnesorge number reciprocal *Z* = 1/Oh in the range of 1 to 10.^29^ Square actuation waveforms were used, and droplet ejection parameters were optimized for stable satellite-free jetting. During the planned experiments stage table was heated up to 80 °C and dispensers located above the stage reach an equilibrium temperature of 34 °C. For this reason, the IPDI dispenser was heated to 40 °C, the PEA 400 has a higher viscosity and the temperature was set to 60 °C. At dispensing temperatures IPDI and PEA 400 have dynamic viscosity of 5.8 mPa s and 6.7 mPas s, respectively (ESI Table S1†). Impulse time was optimized by maximizing droplet volume with set actuation voltage. Afterward actuation voltage was changed to obtain droplets of the required volume. The whole process was monitored by the optical system under stroboscopic illumination (ESI Table S2†). The dispensers were aligned using microscrews so both droplets were ejected in crossing trajectories. The nozzles were located 10 mm above the printing stage and the collision point was approximately 3 mm from the nozzle.

During these tests, pillars with different ink volume ratios were printed (Fig. 4). The slight angling of the pillar is the result of coalesced droplet trajectory being slightly off the surface normal. The pillars were printed at a frequency of *f* = 200 Hz. Due to the maximum height of the pillar being larger than 15 mm the distance between nozzles and the surface was set to 36 mm. After reaching maximum height ∼20 mm the pillar bends at the base, due to insufficient solidification, and the whole structure collapses.

### 2.10. Printing parameters

A solid block was sliced using Slic3R open-source slicer software for FDM printing. The extrusion rate in the software is converted into a droplet ejection signal with 40 drops per 1 mm. The print was carried out with a maximum stage table velocity of 10 mm s^−1^ with a peak droplet generation frequency of 400 Hz. The stage acceleration above 80 mm s^−2^ introduces vibrations on the printhead which causes disruptions in collision and coalescence of the droplets. For this reason, the stage acceleration has been capped at 50 mm s^−2^. Since the contact angle between mixed inks and Teflon surface is unknown it was assumed to be 90°. Distance between threads was then calculated based on estimated coalesced droplet volume ∼940 pL and the number of droplets per thread. Assuming that thread is a half-cylinder with a total volume equal to the volume of the deposited droplets thread diameter equals *d*_th_ = 308 μm. Assuming wetting of the previous thread, to produce a level plane distance between threads takes value *S* = 242 μm. Due to limitations of stage motors distance between threads was set to *S* = 250 μm, which corresponds to a layer height of *h*_L_ = 150 μm. To increase the print speed only 10 layers were printed to a total block dimension of 90 × 10 × 1.5 mm. All the layers were printed along the longer block axis. Samples 1–10 and A–C were printed along printer *X*-axis (within the plane of droplet collision) samples D-F were printed along printer *Y*-axis (perpendicular to the plane of droplet collision).

### 2.11. Printing conditions

The prints were carried out with various stage temperatures from room temperature to 80 °C. Since IPDI reacts with water vapor the whole printer is placed inside an acrylate box flushed with dry air (dehumidified 7.5 bar at 4 °C, RH = 4.6%, *w*(H_2_O) = 667 ppm) to provide an atmosphere with relative humidity below RH < 5%, measured by humidity sensor HYT221 with ± 1.8% precision.

### 2.12. Finished product conditioning

The printed samples were conditioned at the printing stage without changing the stage temperature. After 1 hour the sample was placed in a desiccator for another 6 days for the samples 1–13, or 3 days for the samples A–F.

### 2.13. Printed sample characterization

FT-IR spectra were recorded on a Thermo Scientific Nicolet iS5 spectrometer. Tensile strength tests were conducted by the Instron 5566 Universal Testing Machine, equipped with a 10 kN measuring head and self-tightening roller tensile grips. Tensile strength tests were performed at room temperature at a running rate of 50 mm min^−1^. The Young modulus *E* was calculated based on point range starting at the second point and finishing at the point allowing for a line correlation with a coefficient of determination *R*^2^ ≥ 0.995. Yield point *Y* was calculated as a point on a stress–strain curve with a tangent equal to *E*/2.

## 3. Results and discussion

Reactive inkjet printing is an interesting technology that strives to utilize the chemical nature of inks to produce materials with unique properties. The main objective of this research is to show the possibility of reactive inkjet 3D printing of structure materials. Due to rapid polymerization of diamines with diisocyanates, causing fast solidification of material after mixing at ambient temperatures, polyurea system has been selected. The first test of the reactive ink composition to assess the applicability in micro-reactive inkjet printing is the possibility to print pillars (Fig. 4). Inks capable to produce pillars should exhibit short gel-times therefore produced 3D structures are not deformed by spreading of the mixed reacting material. The ink spreading has been confirmed by Krober *et al.* who used polyol such as PEG instead of PEA, although in such systems polymerization rate can be enhanced using catalyst.^28^ In almost every tested composition the pillar shape is determined by the interplay of two effects: (1) droplet losing velocity as a result of air resistance and as a consequence lowering the impact energy on the surface but reducing deposition precision, (2) droplet showing increased viscosity due to undergoing a chemical reaction. A wider footprint is observed as a result of lower droplet precision (A 1.36 mm; B 1.52 mm; C 1.52 mm, D 1.01 ± 0.02 mm). At the middle of the pillar increased deposition precision results in thinner pillar diameter (A 1.53 mm; B 1.25 mm; C 1.52 mm, D 0.72 ± 0.08 mm). The top of the pillar is again slightly thicker due to higher droplet velocity and lower viscosity which promotes droplet spreading (A 1.84 mm; B 1.76 mm, C 1.78 mm, D 0.69 ± 0.18 mm). Afterward due to a fast increase in pillar height the pillar bends due to insufficient gelling of bottom layers. Pillar printing was stopped as soon as the shifting of the pillar was observed. In principle printing distance as well as droplet kinetic energy could be optimized by this pillar printing method to set printing parameters to obtain the thinnest pillars. This would indicate the lowest material spreading and best deposition precision. Printed pillar circumference is larger than that of 2D printed thread *d*_t_ = 0.3 mm due to mentioned effects *i.e.* droplet spreading on collision with the gelled surface and printhead droplet deposition instability. The droplets collided with impact parameter *X* = 0 (relative velocity vector pointing directly between droplet centers).^30^ The collision energy characterized by Webber Number We for all tested ink proportions did not exceed We = 5. In such conditions for miscible liquids irrespectively of impact parameter full coalescence of the droplets should be expected.^31^ However, due to droplet jetting instability as well as slight deviations in droplet trajectory the reliable droplet coalescence can be observed only when the modulus of impact parameter *X* is lower than |*X*| = 0.7. With further increase of impact parameter *X* probability of coalescence decreases and reaches 0% when impact parameter *X* takes the value of |*X*| = 0.95. Since a slow change in droplet volume and velocity can be observed during longer prints, trigger time must be corrected to obtain reliable droplet coalescence.

**Fig. 5:**
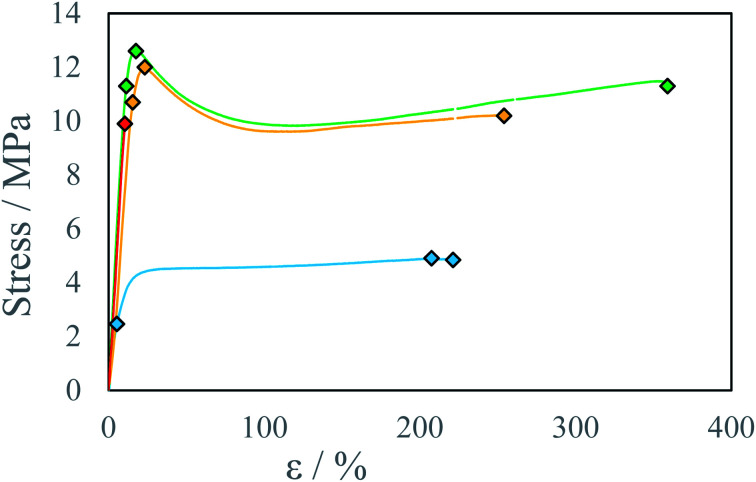
Stress–strain curve for beams printed at the temperature *T* = 80 °C with different IPDI excesses *R*^E^: ● 3%, ● 16%, ● 23%, ● 25%.

**Fig. 6:**
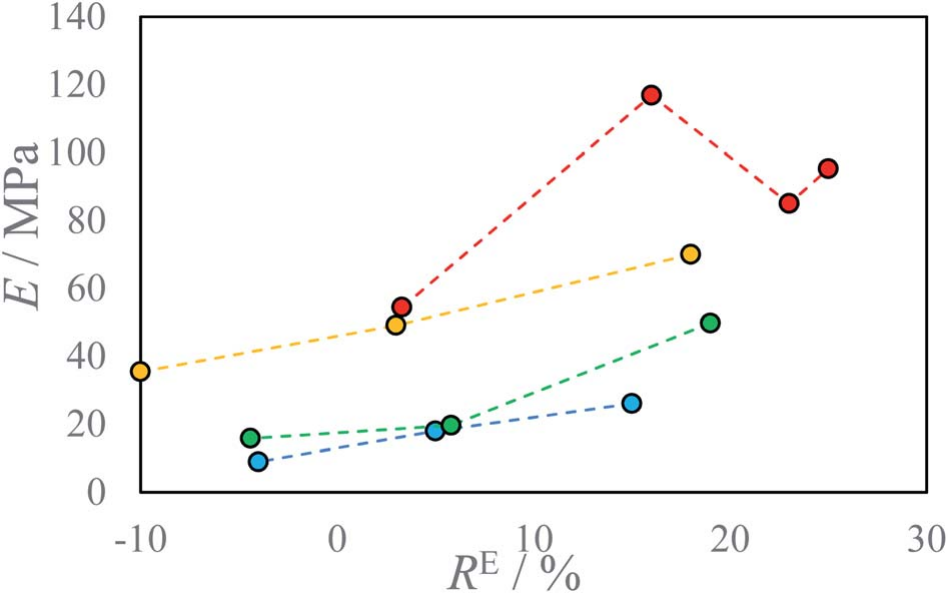
Young modulus *E* of produced materials as a function of IPDI excess *R*^E^ at different temperatures *T*: ● 22 °C, ● 40 °C, ● 60 °C, ● 80 °C.

Several testing beams were printed with varied stage temperatures and inks' ratios to investigate the influence of printing parameters on produced material properties. An increase in IPDI molar excess *R*^E^ results in an increase in stress resistance (Fig. 5 and S5–S7) and an increase in Young modulus *E* (Fig. 6). At elevated temperatures and higher *R*^E^ a change in material characteristics can be observed, from ductile (*R*^E^ = 3%) by quasi-brittle (*R*^E^ = 16% and *R*^E^ = 23%) to rigid in the case of highest IPDI excess (*R*^E^ = 25%). The best stress resistance was observed for the sample printed at *T* = 80 °C and *R*^E^ = 16%. Further increase in *R*^E^ = 23% does not increase stress resistance, but decreases elongation at break and Young modulus *E*. With *R*^E^ = 25% inductile material can be produced and a slight increase in Young modulus *E* can be observed relative to sample printed at *R*^E^ = 23% (Fig. 5). This can be explained by an increase in the number of urea groups in the material which introduce rigidity due to hydrogen bonding. Additionally, urea groups can react with residual isocyanate groups forming a rigid crosslinked material.

**Fig. 7:**
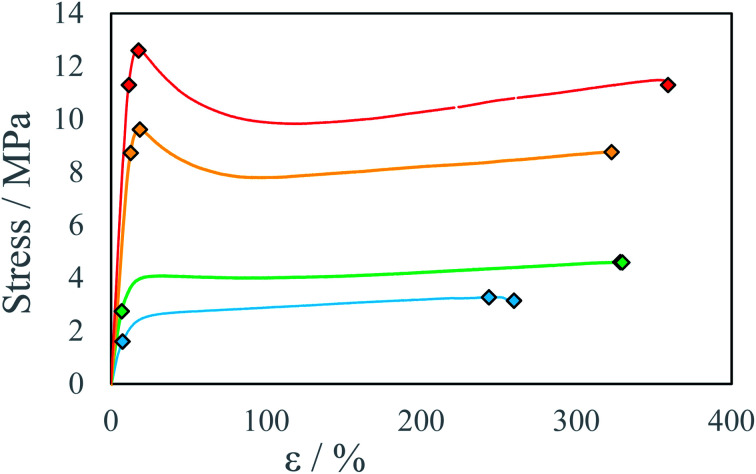
Stress–strain curve for beams printed at IPDI excess *R*^E^ ∼17% at different temperatures *T*: ● 22 °C, ● 40 °C, ● 60 °C, ● 80 °C.

**Fig. 8:**
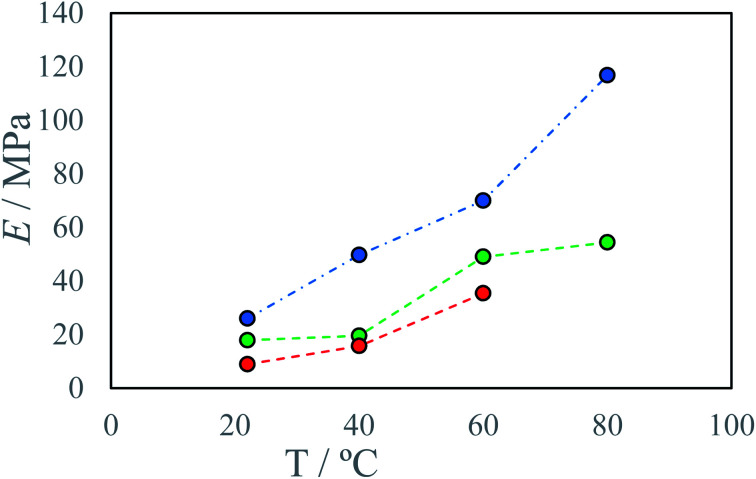
Young modulus *E* of produced materials as a function of process temperature *T* at different IPDI excesses *R*^E^: ● −4%, ● 4%, ● 17%.

In all cases increase in stage temperature increases stress resistance as well as Young Modulus *E* (Fig. 7, 8, S8 and S9 †). The samples were tested up to a temperature of *T* = 80 °C. Further increase in temperature results in the thermal decomposition of IPDI which results in the foaming of the sample. This behavior was further enhanced by increased humidity or water addition into PEA 400 and could be quite interesting in “microfoams” formation for cushioning in MEMS (micro-electro-mechanical systems).^32^ At higher temperatures viscosity of mixed droplets is lower and the diffusion coefficient increases which allows longer chain formation due to substrate availability. The reaction rate also increases with temperature which allows for even longer chain formation due to delay in solidification onset. Additionally, thermal annealing in polyurea systems allows for dynamic crosslinking by bond exchange reactions between unreacted amine and urea group.^33^ Advincula *et al.*^34^ found that thermal annealing at 70 °C for 20 h of polyurea vitrimer increased the ultimate material strength *U* 3-fold from 20 kPa to 60 kPa. This process could further explain increase in stress resistance with increase in process temperature. At elevated temperatures for higher IPDI molar excesses, *R*^E^ a change in stress–strain curve character may be observed from ductile to quasi-rigid material (Fig. 5). In FTIR spectra for samples with IPDI molar excess, a signal can be observed at 2254 cm^−1^ which corresponds to unreacted isocyanate groups (ESI Fig. S10–S28†). This signal is also observed for samples with only slight excess indicating that reaction with water did not occur and unreacted or partially reacted IPDI is still present within the samples (Fig. S29†). This indicates that further material conditioning should change material properties by the creation of urea bonds due to the reaction of residual isocyanates with amines generated by either thermal decomposition of residual isocyanic group or reaction of residual isocyanates with water. Additionally, IPDI present within the samples as well as excess amine could result in self-healing properties of the material by either polyurea polymerization^35^ or bond exchange reactions.^34^

To evaluate process deviation as well as sample maturing time, three beams were printed at the same process parameters and conditioned in a desiccator over 3 days (Fig. S30†). Additional samples were printed to evaluate the anisotropy of prepared materials, by printing longer beam side perpendicular to the plane of collision (Fig. S31†). The estimated relative repeatability of the relevant parameters is 8%, with greater deviations on yield point, up to 25% (Table 3). Lower repeatability of the yield point could be a result of the yield point determination method, not the printing process.

**Fig. 9:**
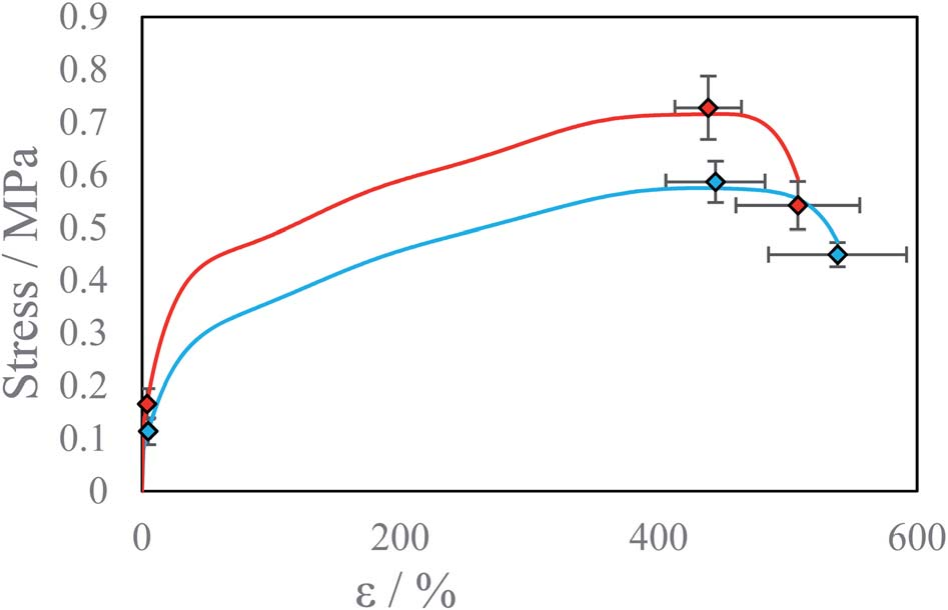
Average stress–strain curve for beams printed along the different axis at IPDI excess *R*^E^ ∼4% and temperature *T* = 40 °C: ● *X*-axis samples A, B, C, ● *Y*-axis samples D, E, F.

Differences in sample properties, printed along or perpendicularly to the plane of droplet collisions are at the level of 20%. Fig. 9 presents average calculated stress–strain curves for samples A, B, C and D, E, F. One reason for the observed anisotropy is lower degree of inter-layer crosslinking reactions due to increased viscosity of gelled reacting material due to lower diffusion coefficient of low-molecular weight substrates.^34^ The second reason is lower droplet deposition precision along the *Y*-axis (perpendicular) which is a result of slight instability of droplet parameters which results in different collision parameter X and different overall momentum of the droplet. As a result, threads printed along the *Y*-axis are wider and have a rougher edge, this influences material properties, but the specific mechanism was not investigated. The bond exchange reaction at elevated temperatures could chemically connect the polymer chains between the printed layers and thereby significantly increase the interlayer adhesion.^34^

**Fig. 10:**
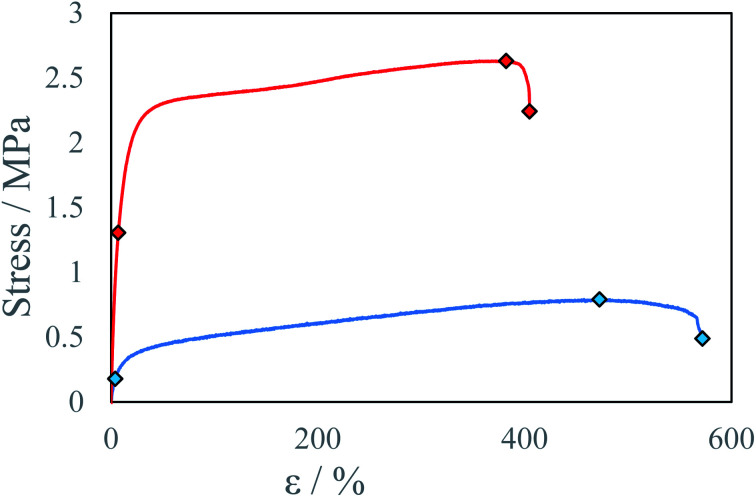
Stress–strain curve for beams with different maturing times at IPDI excess *R*^E^ ∼4% and temperature *T* = 40 °C: ● 6 days, ● 3 days.

In tested samples, ultimate strength *U* increased from 0.8 MPa to 2.6 MPa, and elongation at ultimate strength *ε*_U_ decreased from 472% to 380% for samples conditioned 3 and 6 days respectively (Table 2, Fig. 10). Since in FTIR spectra for both samples show similar intensity of peaks corresponding to the isocyanate group have been observed (Fig. S32†), this behavior cannot be explained by subsequent polymerization reaction between ink components or with water vapor. Polyurethanes, as well as polyureas, exhibit self-organizing properties *via* bond exchange reactions resulting in micro separation of the elastic and rigid phases within the material or entanglement of polyurea chains. Which can play a role during stress tests with strain stiffening behavior.^36^ Sample maturing time has a significant impact on all tested material properties. With longer maturing times increasing material stress resistance and decreasing elongation. The separation of microdomains increases the number of hydrogen bonds formed between urethane or urea groups within the rigid domain thus increasing the stress resistance of the material. Thermal annealing of the samples could shorten maturing times of the samples by increase in bond exchange reaction rate.^34^

**Table 2:** Printing conditions: stage temperature *T*, ink volume ratio PEA 400/IPDI *R*, IPDI molar excess *R*^E^ and material properties: Young modulus *E*, yield Strength *Y*, elongation at yield *ε*_Y_, ultimate strength *U*, elongation at ultimate strength *ε*_U_, strength at break *B*, elongation at break *ε*_B_ of printed samples. Samples 1–13 were conditioned 6 days and samples A–F were conditioned 3 days^a^

No.	T/°C	*R*	*R* ^E^/%	*E*/MPa	*Y*/MPa	*ε*_Y_/%	*U*/MPa	*ε*_U_/%	*B*/MPa	*ε*_B_/%
1	22^b^	1.82	16	26.0	1.60	7.4	3.28	244	3.15	260
2	22^b^	2.20	−4	8.96	0.51	7.4	0.96	244	0.70	382
3	22^b^	2.01	5	17.9	0.66	4.9	1.78	369	1.66	413
4	40	1.78	19	49.7	2.75	6.8	4.6	328	4.59	330
5	60	1.79	18	70.0	8.73	12.7	9.62	18.6	8.76	323
6	40	2.00	6	19.6	1.31	6.9	2.63	382	2.24	405
7	60	2.05	3	49.1	2.32	6.1	4.11	242	3.68	260
8	80	2.05	3	54.4	2.47	5.4	4.91	207	4.85	221
9	40	2.21	−4.4	15.7	0.51	4.4	1.30	194	0.93	284
10	60	2.33	−10	35.4	1.09	4.3	2.82	446	2.56	461
11	80	1.81	16	116.8	11.3	11.4	12.6	17.6	11.3	359
12	80	1.72	23	85.0	10.7	15.5	12.0	23.3	10.2	254
13	80	1.69	25	95.3	—	—	9.9	10.5	9.9	10.5
A	40	2.12	0	5.8	0.18	4.0	0.79	472	0.49	572
B	40	2.03	4.3	5.3	0.13	3.1	0.65	432	0.54	495
C	40	2.06	2.5	5.1	0.19	4.7	0.74	410	0.60	457
D	40	2.01	4.9	2.3	0.13	6.5	0.56	410	0.44	510
E	40	2.03	4.1	3.1	0.13	5.1	0.64	498	0.48	613
F	40	2.00	5.8	4.4	0.08	2.4	0.56	424	0.43	492

a Standard uncertainties *u* are as follows: *u*(*T*) = 0.5 °C.

b *u*(*T*) = 2 °C.

**Table 3:** Average material properties of samples printed within *X* or perpendicularly *Y* to the plane of droplet coalescence

Direction	*E*/MPa	*Y*/MPa	*ε*_Y_/%	*U*/MPa	*ε*_U_/%	*B*/MPa	*ε*_B_/%
*X*	5.4 ± 0.3	0.17 ± 0.03	3.9 ± 0.7	0.73 ± 0.06	438 ± 26	0.54 ± 0.05	508 ± 48
*Y*	3.3 ± 0.9	0.11 ± 0.03	4.7 ± 1.7	0.59 ± 0.04	444 ± 38	0.45 ± 0.02	538 ± 53

## 4. Conclusions

In summary, we have demonstrated that micro-reactive inkjet printing allows for the fabrication of 3D polyurea structures. The reported technique is based on in-flight mixing of generated primary droplets of reactive inks and subsequent deposition on the substrate. In-flight merging of the droplets is controlled by a developed closed-loop control system that corrects trigger time delay to sustain droplet coalescence. Performed test prints of PU showed a clear influence of process parameters on the material properties of printed beams. Printed material shows anisotropic behavior of properties at the level of 20%, although process parameters were not optimized to minimalize material anisotropy. Additionally, it is possible to vary the properties of produced structures by a change in reactive ink ratio.

This way a 3D printer can be conceptualized as a synthesis robot capable of printing versatile patterns with different reactive inks to form the desired product as a result of chemical reactions. By online control in droplet volume and reactant proportions, it is possible to produce functionally graded materials.

## Author contributions

Maciej Zawadzki: conceptualization, methodology, formal analysis, writing original draft as well as review & editing, visualization, supervision, project administration. Krzysztof Zawada: methodology, investigation, validation, and data curation. Sebastian Kowalczyk and Andrzej Plichta: resources, investigation, writing – original draft. Jan Jaczewski and Tomasz Zabielski: methodology, software, writing – original draft.

## Conflicts of interest

There are no conflicts to declare.

## Supplementary Material

RA-012-D1RA07883F-s001

## References

[cit1] Ngo T. D., Kashani A., Imbalzano G., Nguyen K. T. Q., Hui D. (2018). “Additive manufacturing (3D printing): a review of materials, methods, applications and challenges”. Composites, Part B.

[cit2] Masood S. H. (1996). “Intelligent rapid prototyping with fused deposition modelling”. Rapid Prototyp. J..

[cit3] Agarwala M., Bourell D., Joseph B., Marcus H., Barlow J. (1995). “Direct selective laser sintering of metals”. Rapid Prototyp. J..

[cit4] https://www.hp.com/pl/pl/printers/3d-printers/products/multi-jet-technology.html

[cit5] Schultz A. R., Lambert P. M., Chartrain N. A., Ruohoniemi D. M., Zhang Z., Jangu C., Zhang M., Williams C. B., Long T. E. (2014). “3D Printing Phosphonium Ionic Liquid Networks with Mask Projection Microstereolithography”. ACS Macro Lett..

[cit6] Dendukuri D., Pregibon D. C., Collins J., Hatton T. A., Doyle P. S. (2006). “Continuous-flow lithography for high-throughput microparticle synthesis”. Nat. Mater..

[cit7] Macdonald N. P., Cabot J. M., Smejkal P., Guijt R. M., Paull B., Breadmore M. C. (2017). “Comparing Microfluidic Performance of Three-Dimensional (3D) Printing Platforms”. Anal. Chem..

[cit8] Kelly B. E., Bhattacharya I., Heidari H., Shusteff M., Spadaccini C. M., Taylor H. K. (2019). “Volumetric additive manufacturing *via* tomographic reconstruction”. Science.

[cit9] https://www.mvpind.com/product/reactive-additive-manufacturing/

[cit10] Löffelmann U., Wang N., Mager D., Smith P. J., Korvink J. G. (2012). “Solvent-free inkjet printing process for the fabrication of conductive, transparent, and flexible ionic liquid-polymer gel structures”. J. Polym. Sci., Part B: Polym. Phys..

[cit11] Singh M., Haverinen H. M., Dhagat P., Jabbour G. E. (2010). “Inkjet Printing—Process and Its Applications”. Adv. Mater..

[cit12] Chiolerio A., Bocchini S., Porro S. (2014). “Inkjet Printed Negative Supercapacitors: Synthesis of Polyaniline-Based Inks, Doping Agent Effect, and Advanced Electronic Devices Applications”. Adv. Funct. Mater..

[cit13] Singh G., Verma A. (2017). A Brief Review on injection moulding manufacturing process”. Mater. Today: Proc..

[cit14] Krainer S., Smit C., Hirn U. (2019). “The effect of viscosity and surface tension on inkjet printed picoliter dots”. RSC Adv..

[cit15] Al-Milaji K. N., Secondo R. R., Ng T. N., Kinsey N., Zhao H. (2018). Interfacial Self-Assembly of Colloidal Nanoparticles in Dual-Droplet Inkjet Printing. Adv. Mater. Interfaces.

[cit16] Abulikemu M., Husni Da'as E., Haverinen H., Cha D., Malik M. A., Jabbour G. E. (2014). “InSituSynthesis of Self -AssembledGold Nanoparticles on Glass or Silicon Substrates through Reactive Inkjet Printing”. Angew. Chem..

[cit17] Jeon S., Park S., Nam J., Kang Y., Kim J.-M. (2016). “Creating Patterned Conjugated Polymer Images Using Water-Compatible Reactive Inkjet Printing”. ACS Appl. Mater. Interfaces.

[cit18] Sykes T. C., Castrejón-Pita A. A., Rafael Castrejón-Pita J., Harbottle D., Khatir Z., Thompson H. M., Wilson M. C. T. (2020). “Surface jets and internal mixing during the coalescence of impacting and sessile droplets”. Phys. Rev. Fluids.

[cit19] Craig S., Tuck C. J., Ashcroft I. A., Wildman R. D. (2017). “3D reactive inkjet printing of polydimethylsiloxane”. J. Mater. Chem. C.

[cit20] Stempien Z., Rybicki T., Rybicki E., Kozanecki M., Szynkowska M. I. (2015). “*In situ* deposition of polyaniline and polypyrrole electroconductive layers on textile surfaces by the reactive ink-jet printing technique”. Synth. Met..

[cit21] Rios O., Carter W., Post B., Lloyd P., Fenn D., Kutchko C., Rock R., Olson K., Compton B. (2018). “3D printing *via* ambient reactive extrusion”. Mater. Today Commun..

[cit22] Hinterbichler H., Planchette C., Brenn G. (2015). “Ternary drop collisions”. Exp. Fluids.

[cit23] Teo M. Y., Stuart L., Aw K. C., Stringer J. (2017). “Micro-reactive Inkjet Printing of Three-Dimensional Hydrogel Structures”. MRS Adv..

[cit24] Teo M. Y., Kee S., RaviChandran N., Stuart L., Aw K. C., Stringer J. (2020). “Enabling Free-Standing 3D Hydrogel Microstructures with Microreactive Inkjet Printing”. ACS Appl. Mater. Interfaces.

[cit25] Teo M. Y., Stuart L., Devaraj H., Liu C. Y., Aw K. C., Stringer J. (2019). “The *in situ* synthesis of conductive polyaniline patterns using micro-reactive inkjet printing”. J. Mater. Chem. C.

[cit26] Teo M. Y., RaviChandran N., Kim N., Kee S., Stuart L., Aw K. C., Stringer J. (2019). “Direct Patterning of Highly Conductive PEDOT:PSS/Ionic Liquid Hydrogel *via* Microreactive Inkjet Printing”. ACS Appl. Mater. Interfaces.

[cit27] Teo M. Y., Kee S., Stuart L., Stringer J., Aw K. C. (2021). “Printing of covalent organic frameworks using multi-material in-air coalescence inkjet printing technique”. J. Mater. Chem. C.

[cit28] Krober P., Delaney J. T., Perelaer J., Schubert U. S. (2009). “Reactive inkjet -printing of polyurethanes”. J. Mater. Chem..

[cit29] Derby B. (2010). “Inkjet Printing of Functional and Structural Materials: Fluid Property Requirements, Feature Stability, and Resolution”. Annu. Rev. Mater. Res..

[cit30] Chen R.-H., Chen C.-T. (2006). “Collision between immiscible drops with large surface tension difference: diesel oil and water”. Exp. Fluids.

[cit31] Gao T. C., Chen R.-H., Pu J., Lin T. (2005). “Collision between an ethanol drop and a water drop”. Exp. Fluids.

[cit32] Schuster F., Hirth T., Weber A. (2019). “Reactive inkjet printing of polyethylene glycol and isocyanate based inks to create porous polyurethane structures”. J. Appl. Polym. Sci..

[cit33] Lessard J. J., Garcia L. F., Easterling C. P., Sims M. B., Bentz K. C., Arencibia S., Savin D. A., Sumerlin B. S. (2019). “Catalyst-Free Vitrimers from Vinyl Polymers”. Macromolecules.

[cit34] Niu W., Zhang Z., Chen Q., Cao P.-F., Advincula R. C. (2021). “Highly Recyclable, Mechanically Isotropic and Healable 3D-Printed Elastomers *via* Polyurea Vitrimers”. ACS Mater. Lett..

[cit35] Shi Z., Kang J., Zhang L. (2020). “Water-Enabled Room-Temperature Self-Healing and Recyclable Polyurea Materials with Super-Strong Strength, Toughness, and Large Stretchability”. ACS Appl. Mater. Interfaces.

[cit36] Zagar G., Onck P. R., van der Giessen E. (2015). “Two Fundamental Mechanisms Govern the Stiffening of Cross-linked Networks”. Biophys. J..

