# Supplementary material for: 3D reactive inkjet printing of aliphatic polyureas using in-air coalescence technique
Source: RSC Adv. 2022 Jan 25;12(6):3406–15. doi: 10.1039/d1ra07883f (PMC8979265; doi:10.1039/d1ra07883f)

Electronic Supplementary Material (ESI) for RSC Advances.
This journal is © The Royal Society of Chemistry 2022

RSC Advances

SUPPLEMENTARY INFORMATION

3D reactive inkjet printing of aliphatic polyureas using in-air coalescence technique

Maciej Zawadzki[a,b], Krzysztof Zawada[b], Sebastian Kowalczyk[a], Andrzej Plichta[a], Jan Jaczewski[c], Tomasz Zabielski[c]

[a] *Faculty of Chemistry, Warsaw University of Technology, Noakowskiego 3, 00-664 Warsaw, Poland*

[b] *Zdalny Serwis sp z o.o., Wysowska 12, 02-928 Warsaw, Poland*

[c] *AVICON Advanced Vision Control, Jerozolimskie 202, Warsaw, Poland*

* To whom the correspondence should be addressed, e-mail: mzawadzki@ch.pw.edu.pl,

Phone: +48 (22) 234 7475

Table S1.

Measured temperature dependence of dynamic viscosity η and density ρ of reactive inks components

T / ºC	*IPDI*		*PEA 400*	
	ρ / g·cm^{-3}	η / mPa·s	ρ / g·cm^{-3}	η / mPa·s
25	1.05793	10.34	0.96792	24.7
35	1.04998	6.97	0.95979	16.0
40	1.04601	5.87	0.95575	13.2
45	1.04204	4.95	0.95171	10.9
55	1.03411	3.66	0.94364	7.88
60	1.03015	3.16	0.93964	6.69
65	1.02619	2.78	0.93564	5.82
75	1.01826	2.19	0.92765	4.48

Standard uncertainties u are as follows: u(T) = 0.02ºC, $u(\rho)$ = 0.00005 g·cm^{-3}, $u_r(\eta)$ = 5 %

Table S2.

Dispensing parameters impulse time *τ,* impulse voltage *U* and droplet parameters volume *V*, droplet velocity *u* during sample printing.

No.	*IPDI*					*PEA 400*				
	T / ºC	*τ* / *μs*	*U* / V	*V* / pL	*u* / m·s^{-1}	*T* / ºC	*τ* / *μs*	*U* / V	*V* / pL	*u* / m·s^{-1}
1	40	34	68	284±14	2.23	60	79	96	518±15	1.88
2	40	33	67	288±3	1.82	60	77	102	634±15	1.76
3	40	33	67	292±3	1.96	60	80	102	587±9	1.88
4	40	35	65	281±5	1.69	60	77	99	499±11	2.04
5	40	30	75	327±3	2.15	60	75	102	584±18	2.14
6	40	32	82	310±3	2.02	60	77	102	619±11	2.00
7	40	33	73	303±27	2.26	60	72	101	622±14	2.22
8	40	35	70	300±14	2.14	60	73	101	614±13	2.27
9	40	34	73	309±21	2.29	60	70	101	683±50	2.27
10	40	34	70	289±32	2.29	60	68	98	674±53	2.07
11	40	36	65	298±37	2.31	60	98 [a]	71	540±36	2.03
12	40	33	69	276±6	2.00	60	72 [a]	32	473±15	1.89
13	40	313	61	267±3	1.68	60	71 [a]	85	451±4	1.89
A	40	35	64	286±26	1.96	60	70	98	605±35	2.07
B	40	35	63	301±4	2.03	60	71	98	610±38	2.07
C	40	35	63	291±7	2.09	60	70	97	600±27	2.07
D	40	34	62	288±4	1.85	60	69	93	580±23	1.87
E	40	34	62	290±4	1.85	60	69	94	589±12	1.96
F	40	34	63	293±4	1.90	60	69	92	586±30	1.75

[a] dispenser 100 µm

Figure S1

Concept and block design of the micro-reactive printer

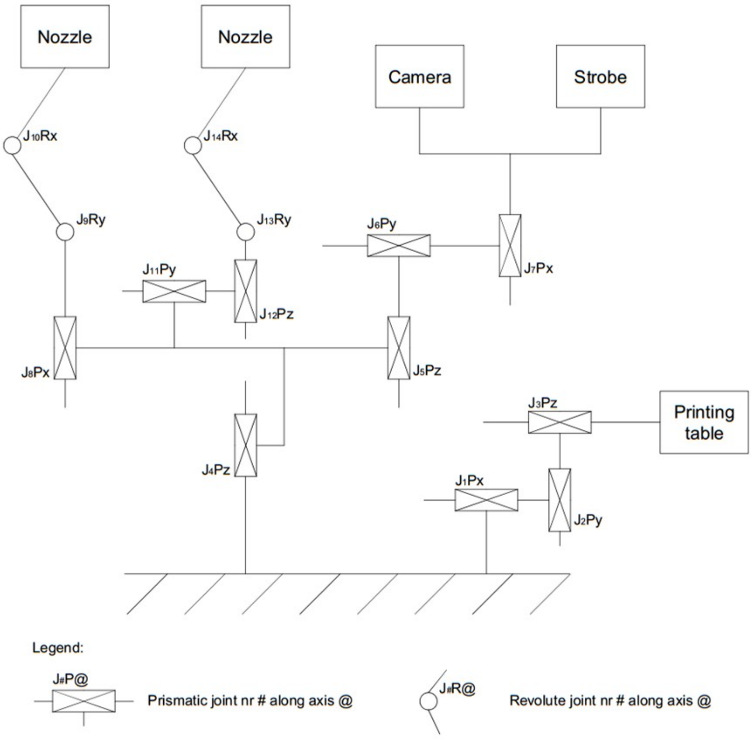

Figure S2

Process control system diagram.

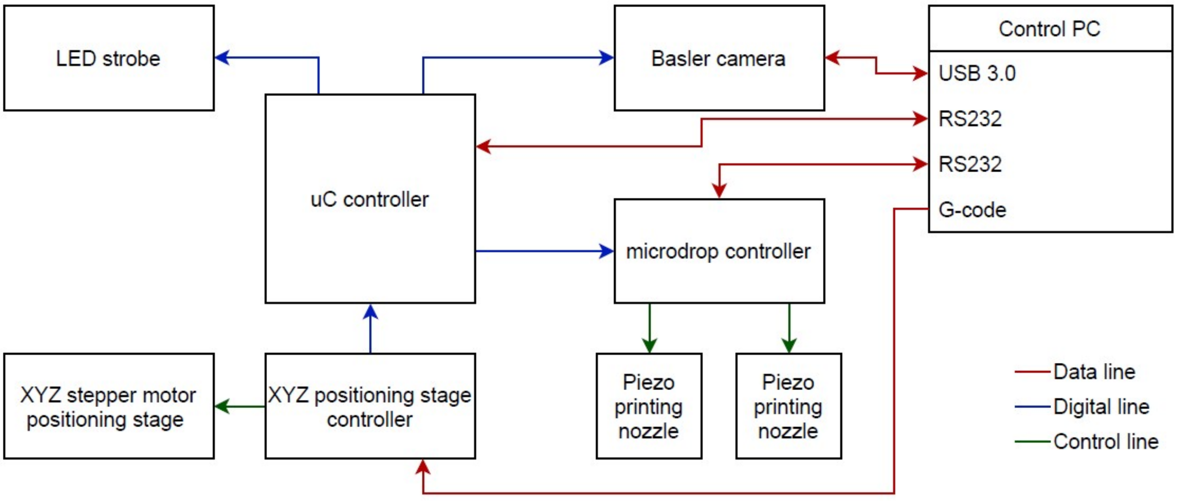

Figure S3

Image acquisition signal diagram.

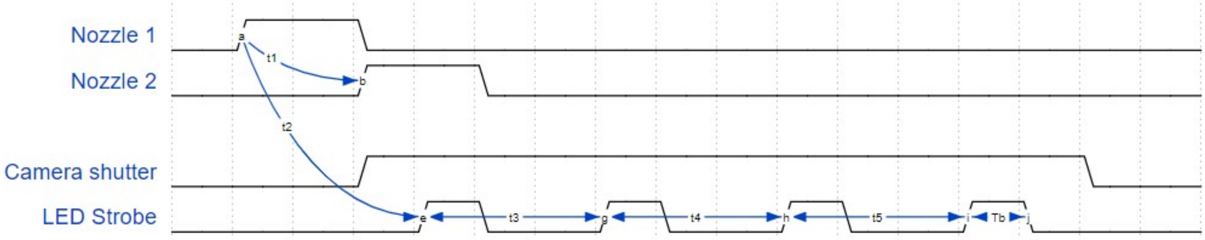

Figure S4

Strobe block diagram.

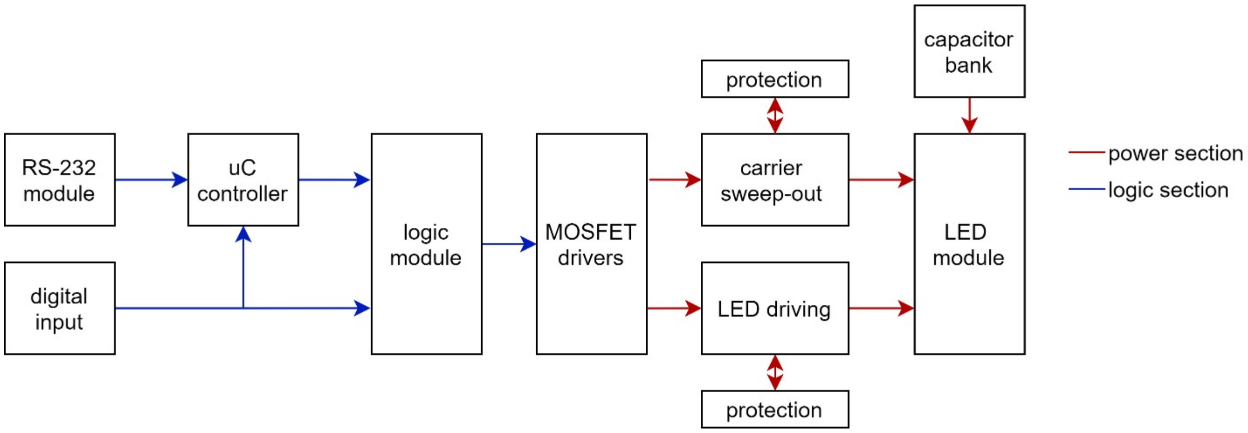

Figure S5

Stress-strain curve for beams printed at room temperature $T = 22°C$ with different IPDI excesses R^E: ● 15%, ● 5%, ● -4%.

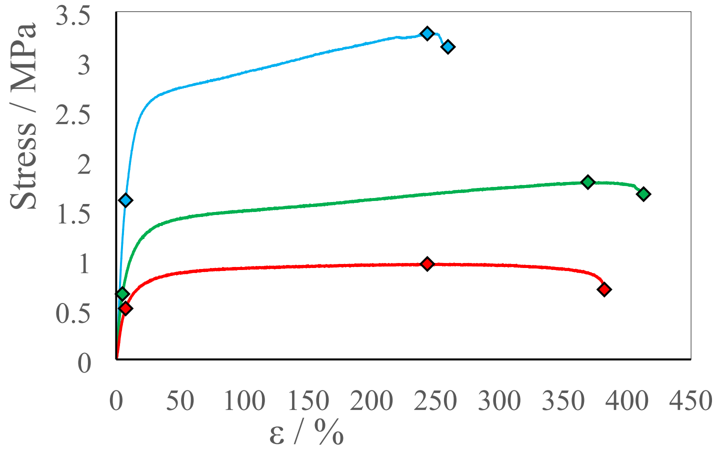

Figure S6

Stress-strain curve for beams printed at the temperature $T = 40°C$ with different IPDI excesses R^E: ● 19%, ● 5.8%, ● -4.4%.

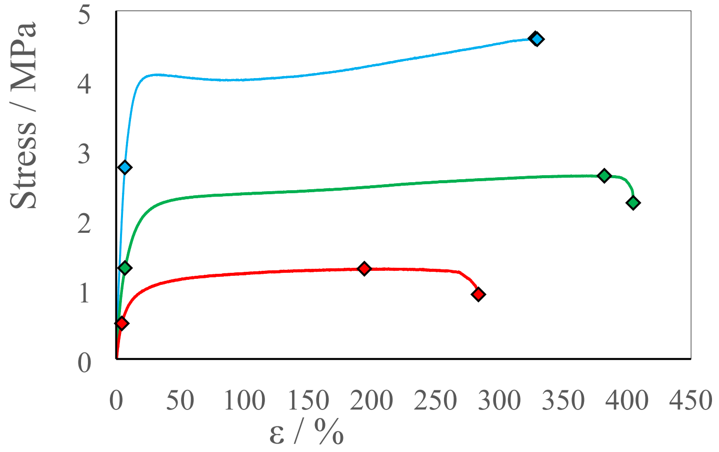

Figure S7

Stress-strain curve for beams printed at the temperature T = 60°C with different IPDI excesses R^E: ● 18%, ● 3%, ● -10%.

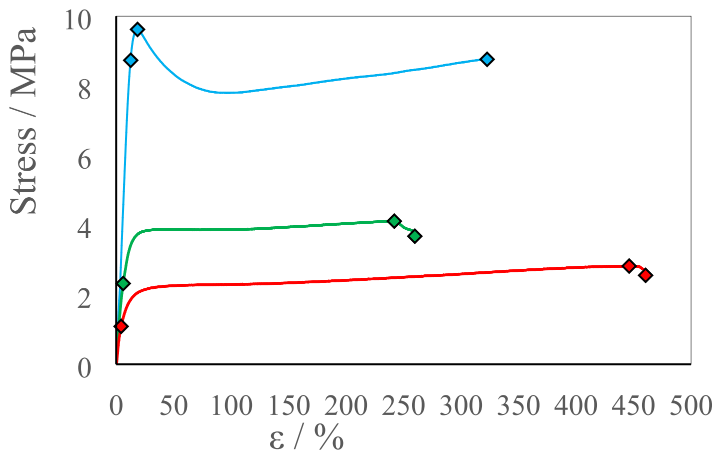

Figure S8

Stress-strain curve for beams printed at IPDI excess $R^E \sim 4\%$ at different temperatures T: ● 22°C, ● 40°C, ● 60°C, ● 80°C

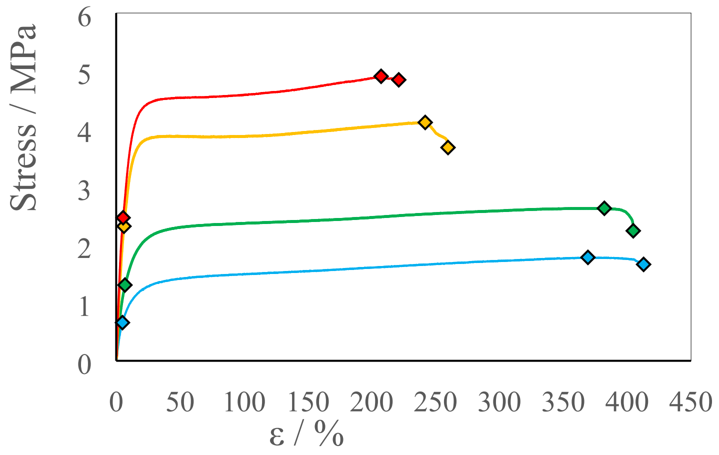

Figure S9

Stress-strain curve for beams printed at IPDI excess R^E ~ - 4% at different temperatures *T*: ● 22°C, ● 40°C, ● 60°C. The beam at T = 60°C was printed with R^E = -10%.

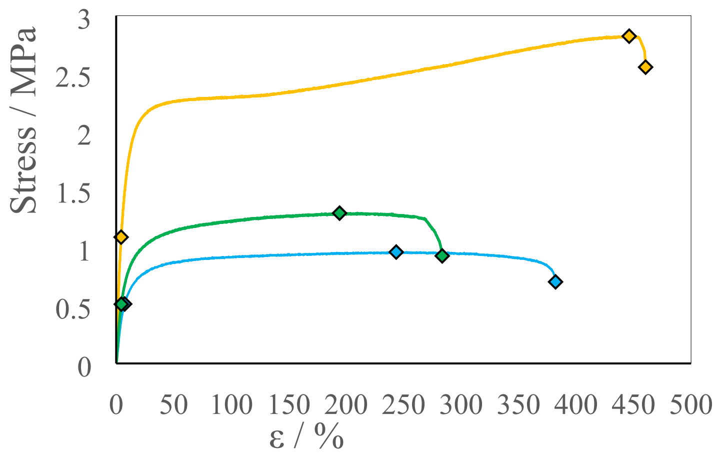

Figure S10

FTIR spectrum of Sample 1

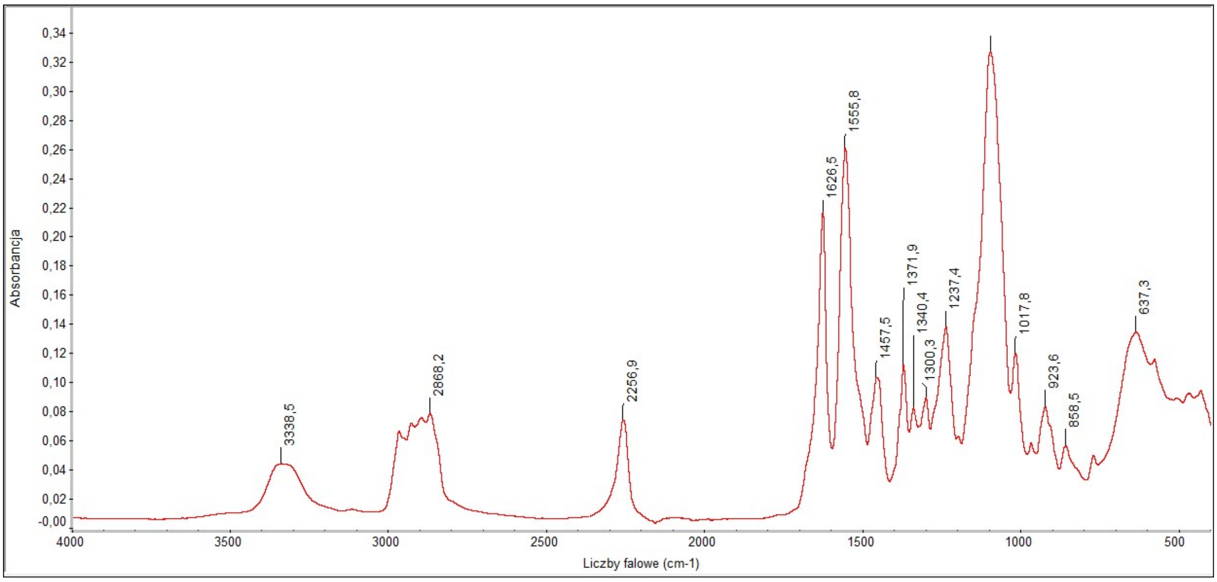

Figure S11

FTIR spectrum of Sample 2

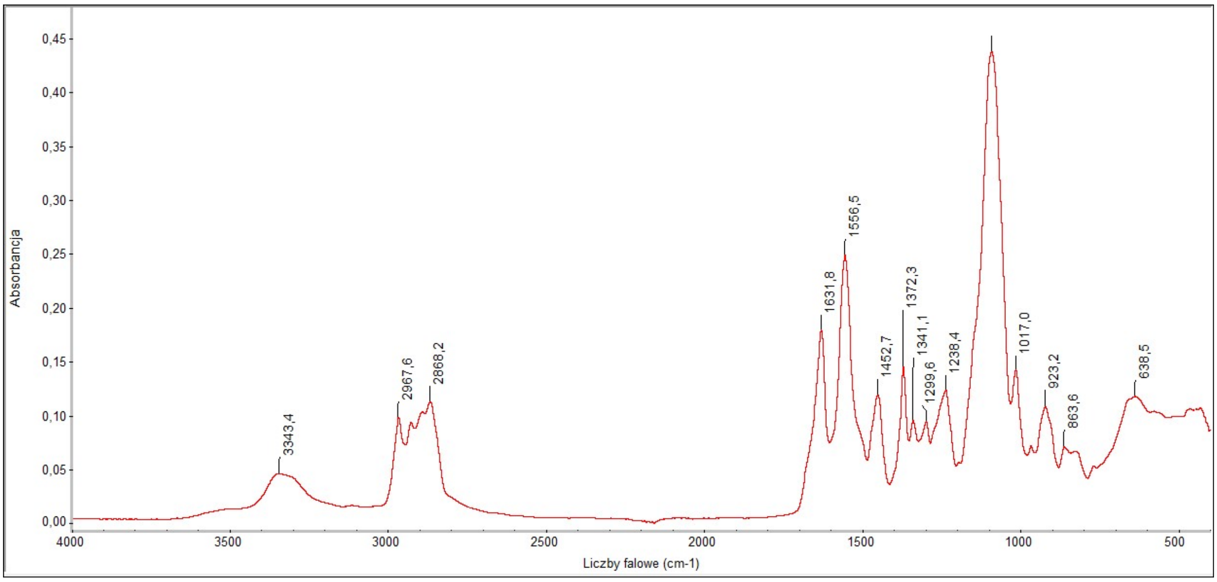

Figure S12

FTIR spectrum of Sample 3

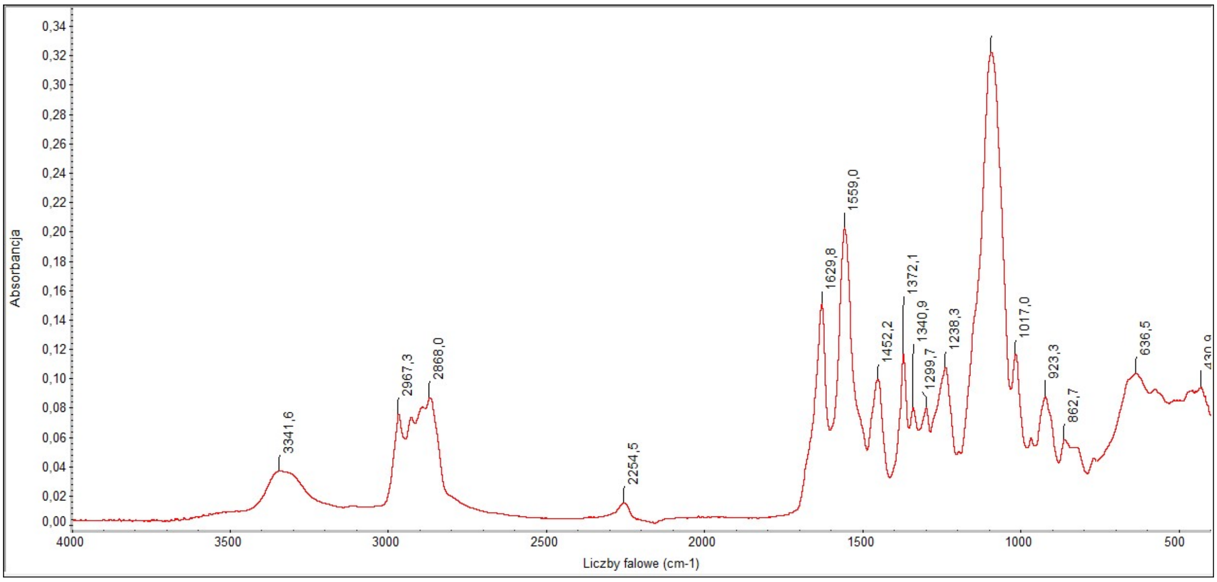

Figure S13

FTIR spectrum of Sample 4

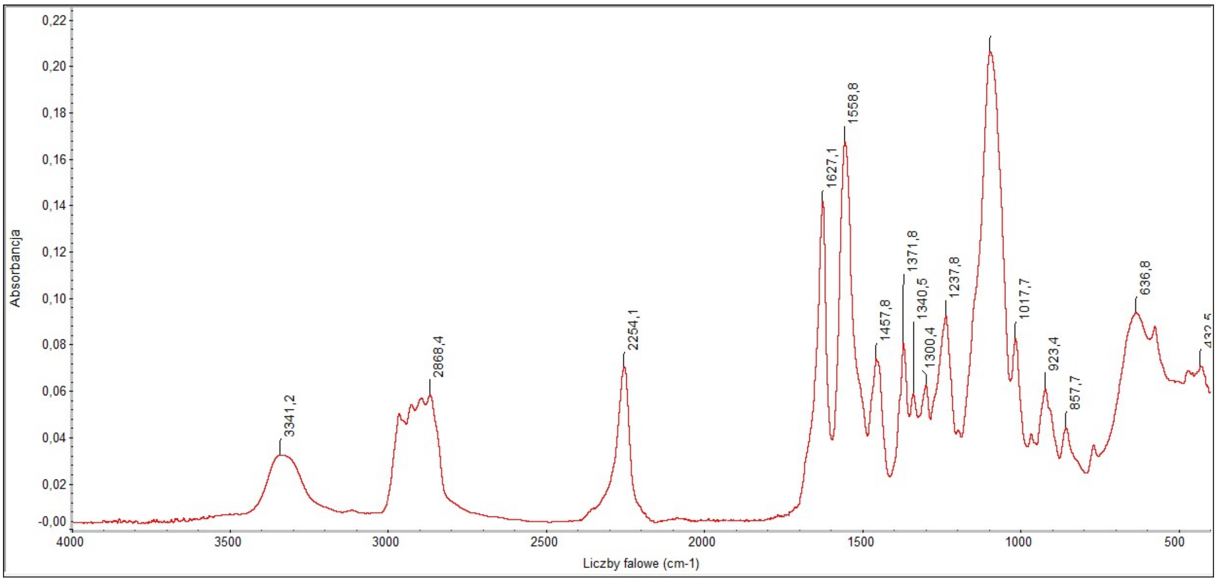

Figure S14

FTIR spectrum of Sample 5

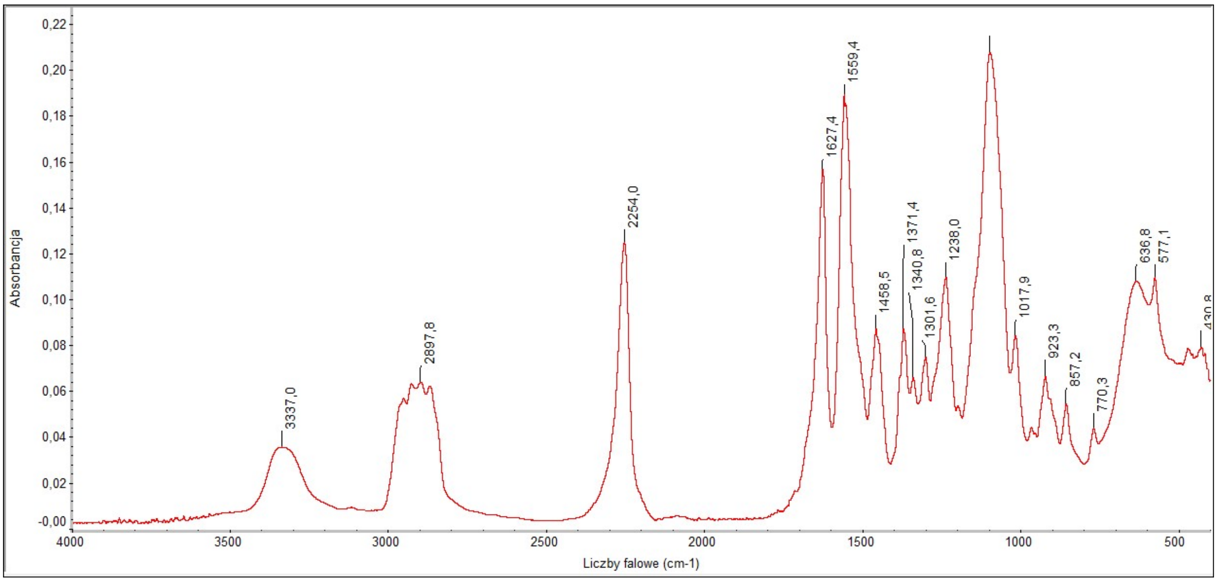

Figure S15

FTIR spectrum of Sample 6

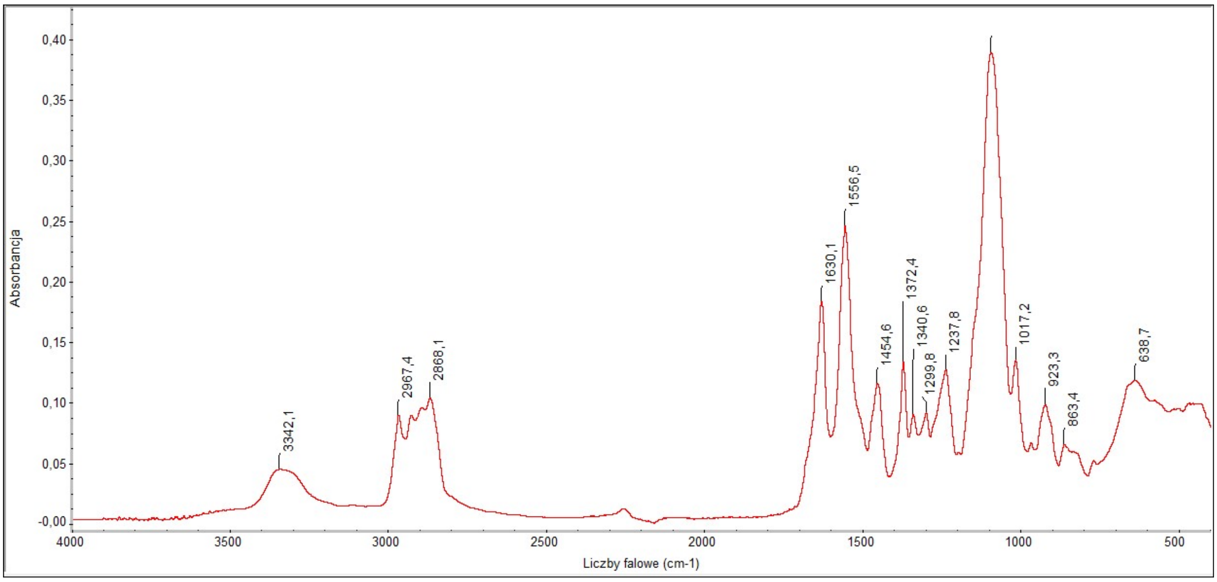

Figure S16

FTIR spectrum of Sample 1=7

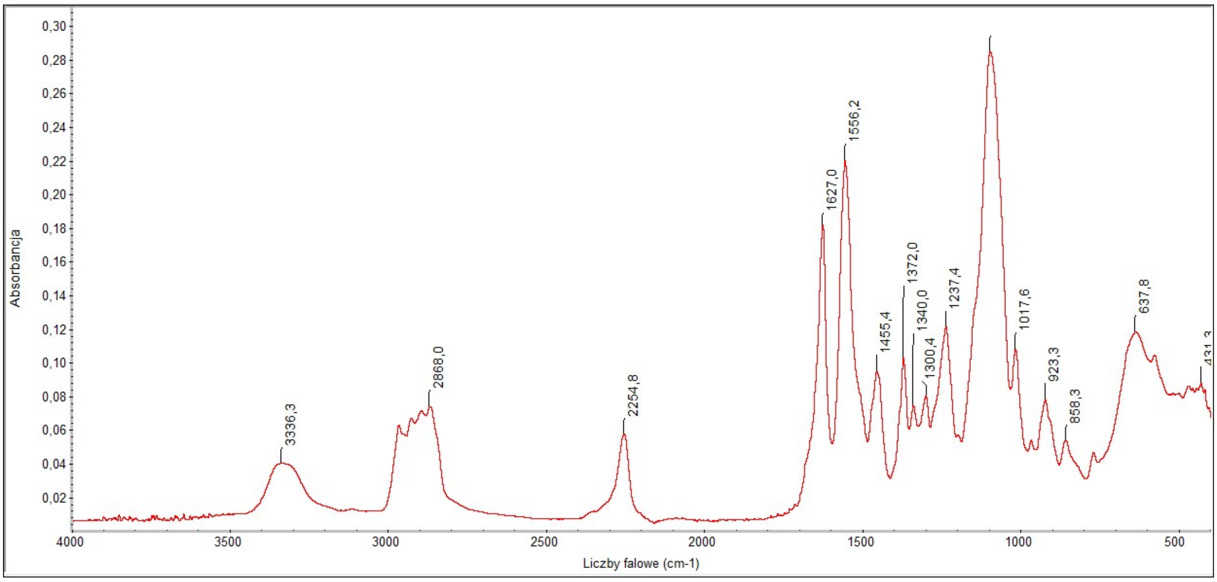

Figure S17

FTIR spectrum of Sample 8

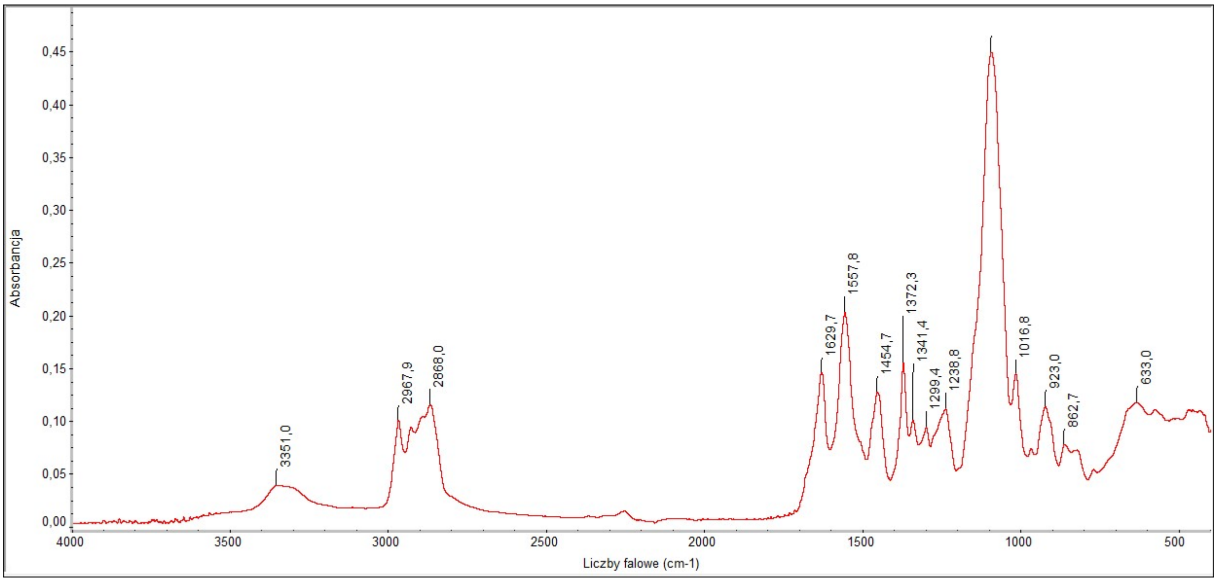

Figure S18

FTIR spectrum of Sample 9

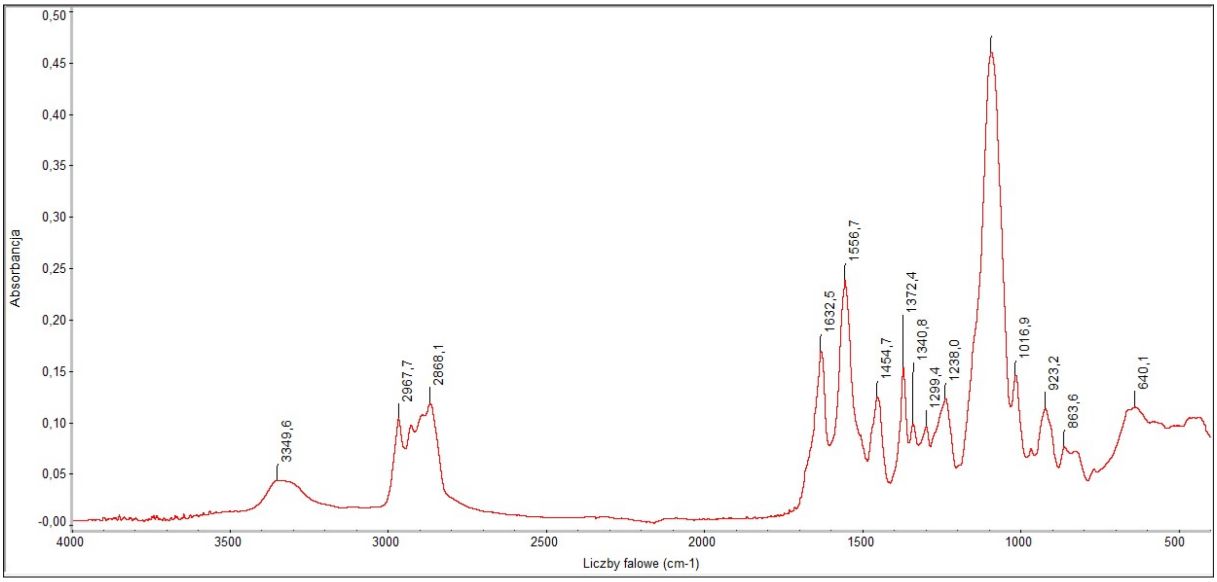

Figure S19

FTIR spectrum of Sample 10

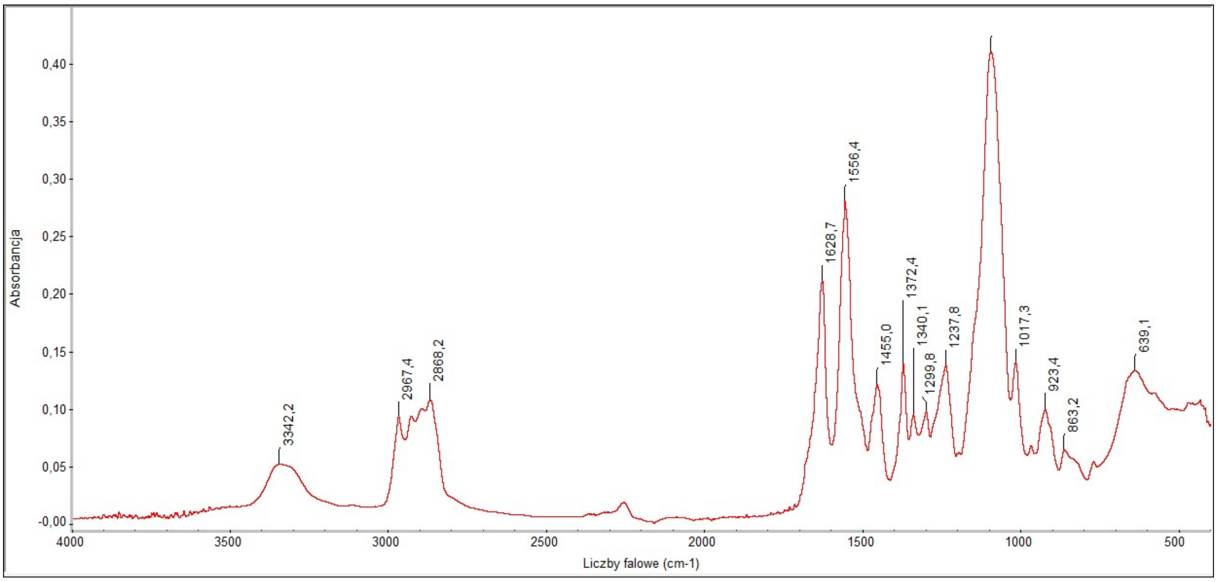

Figure S20

FTIR spectrum of Sample 11

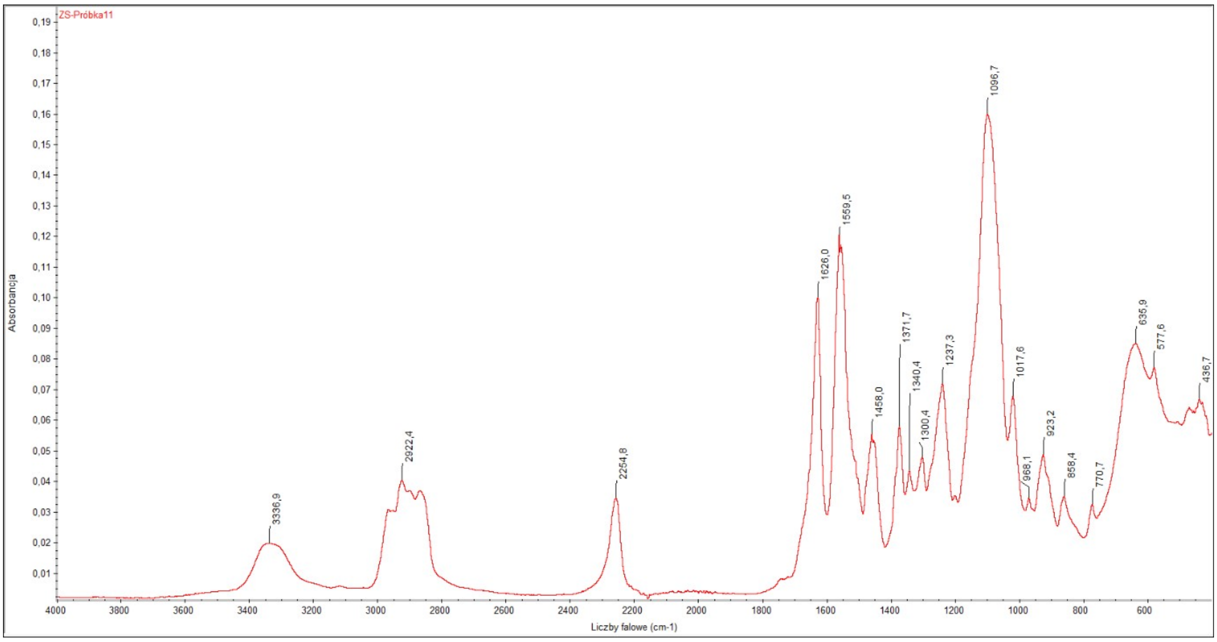

Figure S21

FTIR spectrum of Sample 12

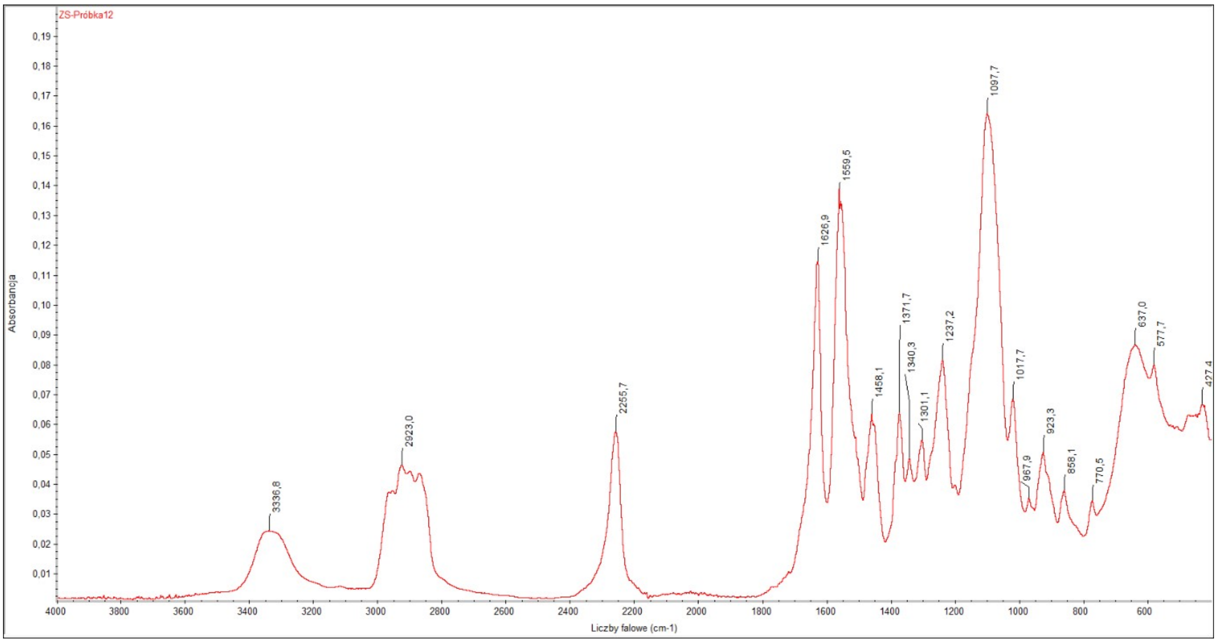

Figure S22

FTIR spectrum of Sample 13

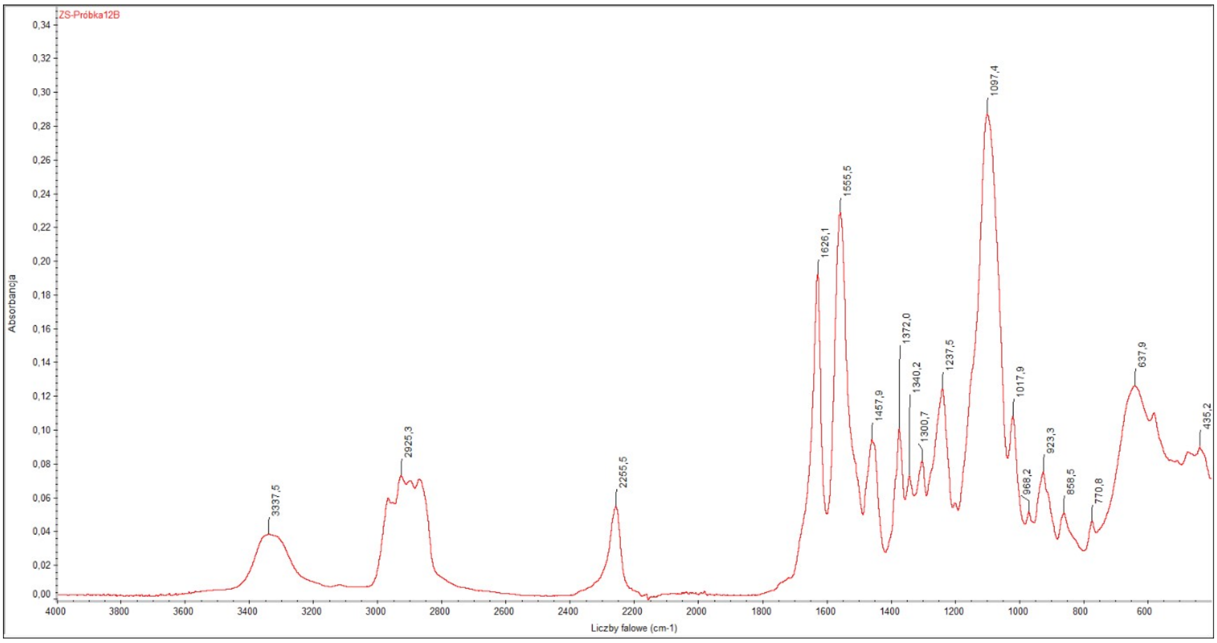

Figure S23

FTIR spectrum of Sample A

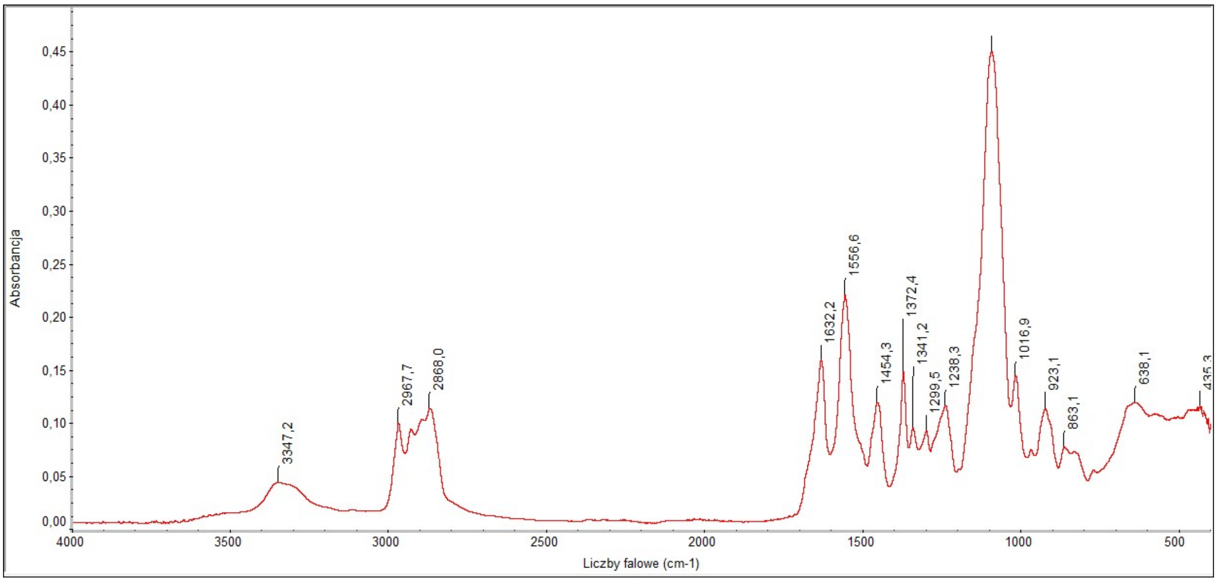

Figure S24

FTIR spectrum of Sample B

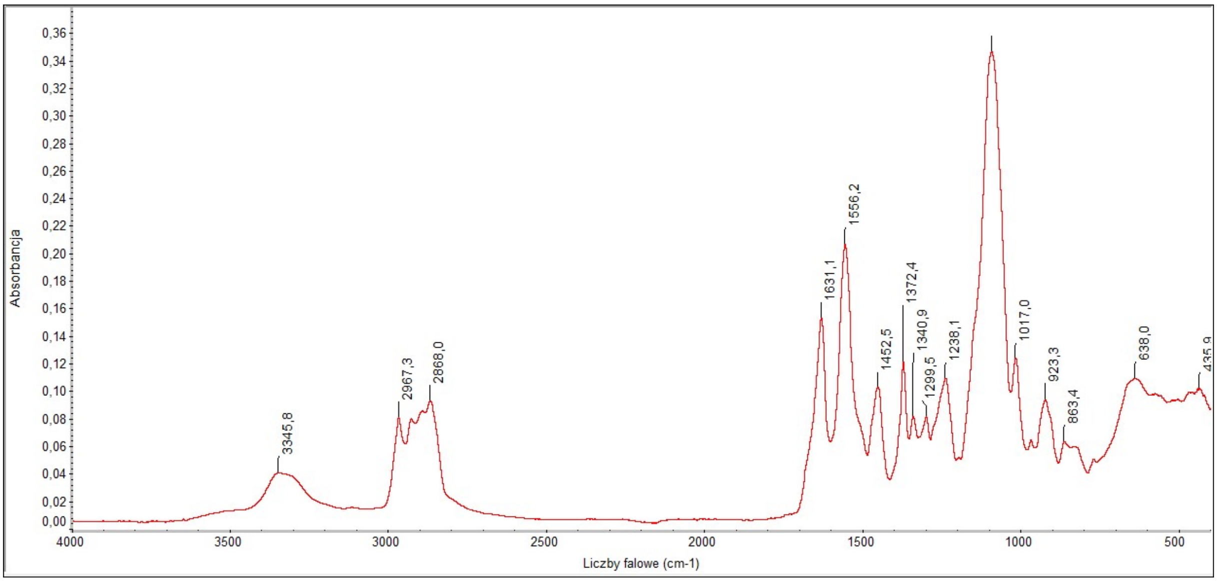

Figure S25

FTIR spectrum of Sample C

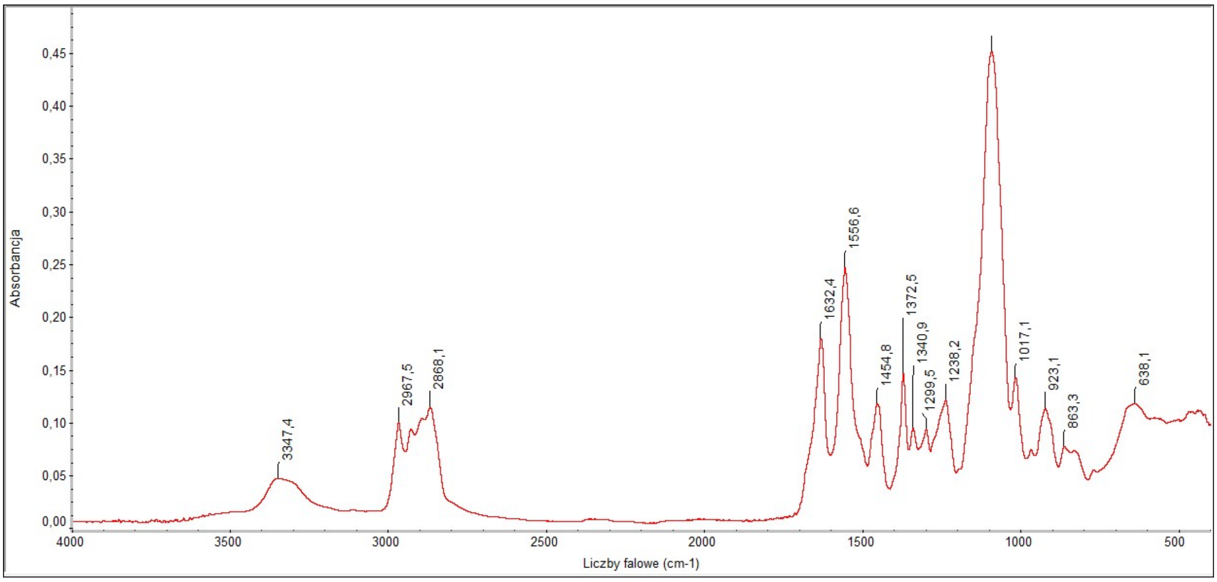

Figure S26

FTIR spectrum of Sample D

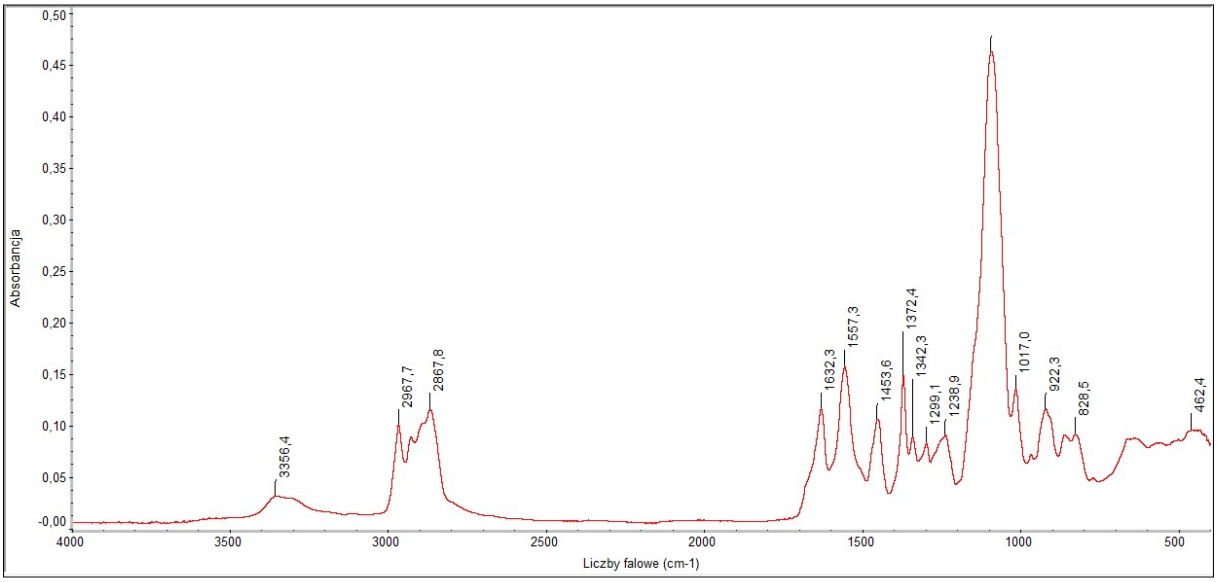

Figure S27

FTIR spectrum of Sample E

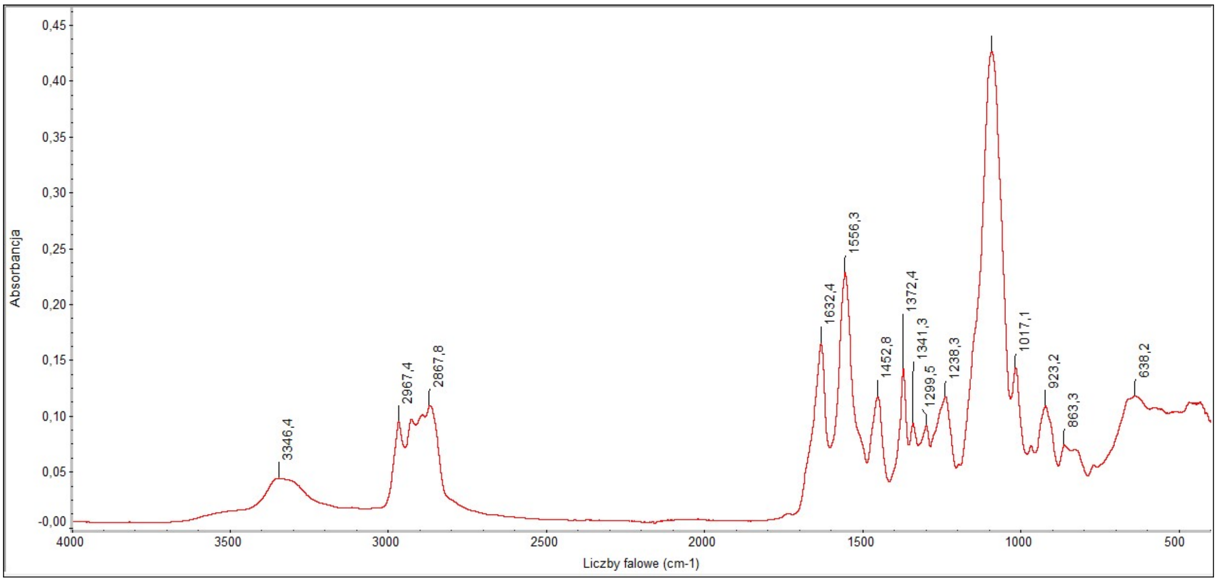

Figure S28

FTIR spectrum of Sample F

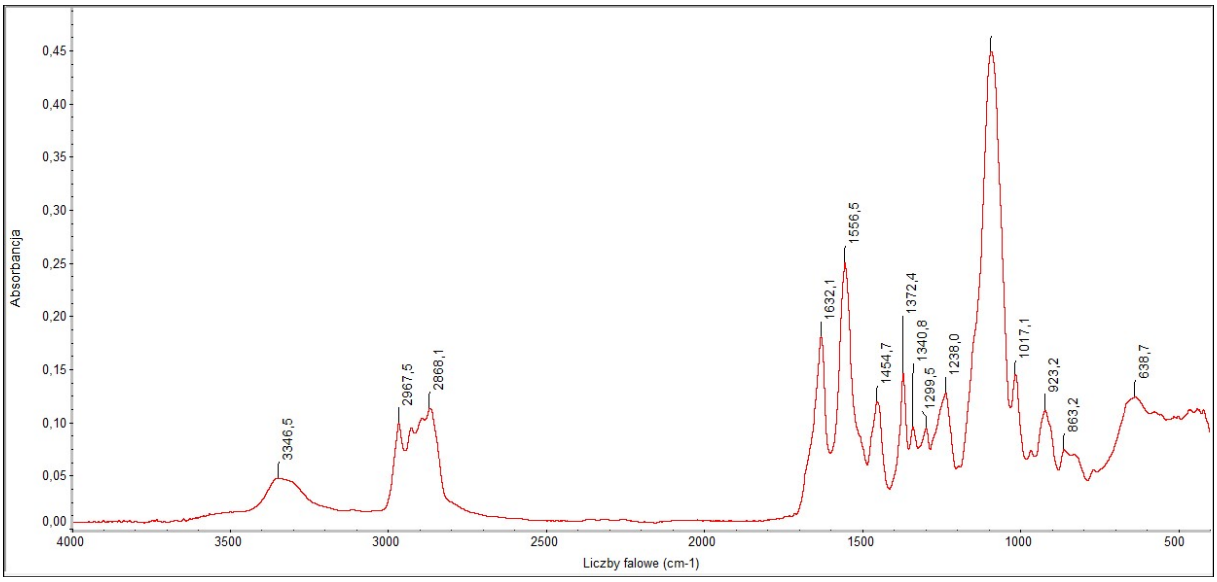

Figure S29

FTIR spectrum of samples printed at temperature T = 40 ºC with different IDPI molar excesses R^E: ● 19%, ● 5.8%, ● -4.4%.

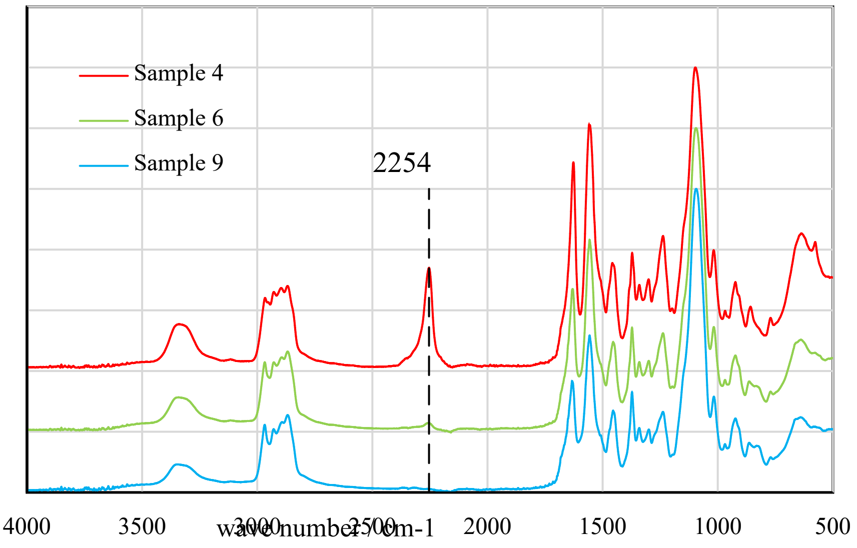

Figure S30

Stress-strain curve for beams printed along the X-axis (within the plane of droplet collision) at IPDI excess R^E ~ 4% and temperature T = 40ºC: ● Sample A, ● Sample B, ● Sample C.

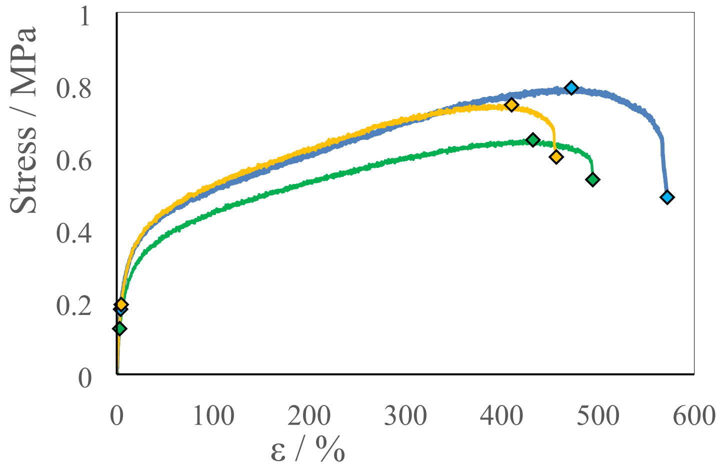

Figure S31

Stress-strain curve for beams printed along the Y-axis (perpendicular to the plane of droplet collision) at IPDI excess R^E ~ 4% and temperature T = 40ºC: ● Sample D, ● Sample E, ● Sample F.

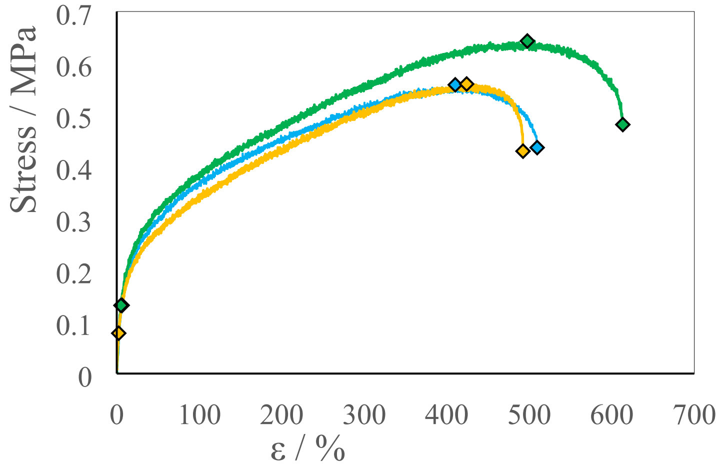

Figure S32

FTIR spectrum of Sample 6 (conditioned for 6 days) and sample F (conditioned 3 days).

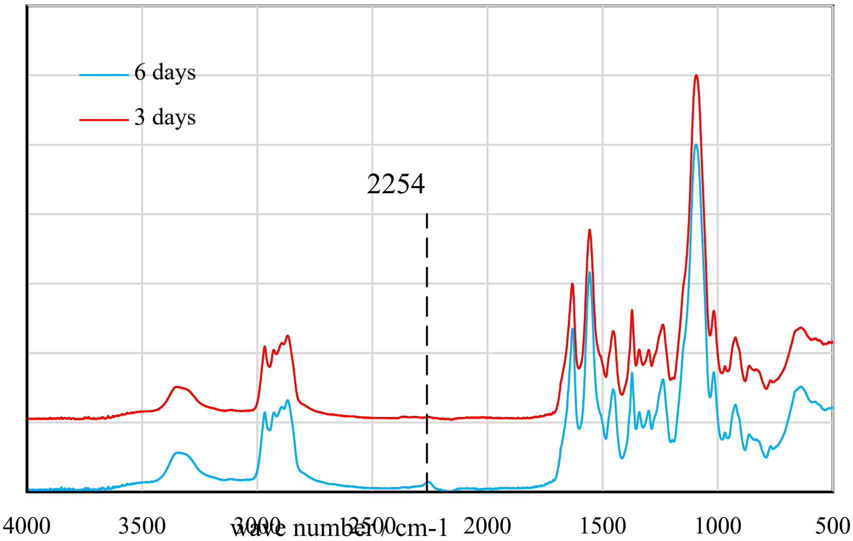

Supplement: RA-012-D1RA07883F-s001 [file RA-012-D1RA07883F-s001.pdf]
